# Shared and divergent pathways for flower abscission are triggered by gibberellic acid and carbon starvation in seedless *Vitis vinifera* L

**DOI:** 10.1186/s12870-016-0722-7

**Published:** 2016-02-01

**Authors:** Sara Domingos, Joana Fino, Vânia Cardoso, Claudia Sánchez, José C. Ramalho, Roberto Larcher, Octávio S. Paulo, Cristina M. Oliveira, Luis F. Goulao

**Affiliations:** Linking Landscape, Environment, Agriculture and Food (LEAF), Instituto Superior de Agronomia (ISA), Universidade de Lisboa (ULisboa), Lisbon, Portugal; Instituto de Investigação Científica Tropical, I.P. (IICT), Lisbon, Portugal; Computational Biology and Population Genomics Group, Centre for Ecology, Evolution and Environmental Changes (cE3c), Faculdade de Ciências, Universidade de Lisboa, Lisbon, Portugal; Instituto Nacional de Investigação Agrária e Veterinária, I.P. (INIAV), Oeiras, Portugal; GeoBioTec, Faculdade de Ciências e Tecnolgia (FCT), Universidade Nova de Lisboa (UNL), Caparica, Portugal; FEM-IASMA, Fondazione Edmund Mach, Istituto Agrario di San Michele all’Adige, San Michele all’Adige, TN Italy; Present address: Colégio Food, Farming and Forestry, Universidade de Lisboa (ULisboa), Lisbon, Portugal

**Keywords:** Flower shedding, Gibberellin, Grapevine, Light reduction, Metabolomics, RNA-Seq

## Abstract

**Background:**

Abscission is a highly coordinated developmental process by which plants control vegetative and reproductive organs load. Aiming at get new insights on flower abscission regulation, changes in the global transcriptome, metabolome and physiology were analyzed in ‘Thompson Seedless’ grapevine (*Vitis vinifera* L.) inflorescences, using gibberellic acid (GAc) spraying and shading as abscission stimuli, applied at bloom.

**Results:**

Natural flower drop rates increased from 63.1 % in non-treated vines to 83 % and 99 % in response to GAc and shade treatments, respectively. Both treatments had a broad effect on inflorescences metabolism. Specific impacts from shade included photosynthesis inhibition, associated nutritional stress, carbon/nitrogen imbalance and cell division repression, whereas GAc spraying induced energetic metabolism simultaneously with induction of nucleotide biosynthesis and carbon metabolism, therefore, disclosing alternative mechanisms to regulate abscission. Regarding secondary metabolism, changes in flavonoid metabolism were the most represented metabolic pathways in the samples collected following GAc treatment while phenylpropanoid and stilbenoid related pathways were predominantly affected in the inflorescences by the shade treatment. However, both GAc and shade treated inflorescences revealed also shared pathways, that involved the regulation of putrescine catabolism, the repression of gibberellin biosynthesis, the induction of auxin biosynthesis and the activation of ethylene signaling pathways and antioxidant mechanisms, although often the quantitative changes occurred on specific transcripts and metabolites of the pathways.

**Conclusions:**

Globally, the results suggest that chemical and environmental cues induced contrasting effects on inflorescence metabolism, triggering flower abscission by different mechanisms and pinpointing the participation of novel abscission regulators. Grapevine showed to be considered a valid model to study molecular pathways of flower abscission competence acquisition, noticeably responding to independent stimuli.

**Electronic supplementary material:**

The online version of this article (doi:10.1186/s12870-016-0722-7) contains supplementary material, which is available to authorized users.

## Background

Abscission is the developmental mechanism by which plants are able to shed damaged and excessively formed organs, regulating the metabolic energy required to successfully attain the formation of vegetative and reproductive structures [1]. Abscission encompasses a complex but precise regulation of cell separation that occurs in a specific layer of specialized cells known as abscission zone (AZ) and is simultaneously activated by and responsive to endogenous and exogenous signals, such as abiotic and biotic interactions or exposure to chemical molecules [2, 3]. Once the AZ is properly differentiated, AZ cells acquire competence to respond to triggering-abscission signals through hormone-mediated pathways. After the activation phase, by modulating the expression of genes involved, among others, in cell wall (CW) remodeling and protein metabolism, and a high number of transcription factors, cell separation and differentiation of a protective layer on the proximal side after organ detachment advance as last steps of the abscission process [4, 5]. According to the currently accepted model, the endogenous flow level of inhibitory auxin in an organ destined to abscise must drop to acquire sensitivity to ethylene [6, 7]. Abscisic acid (ABA) is involved by acting as modulator of 1-aminocyclopropane-1-carboxylic acid (ACC) levels, and therefore of ethylene biosynthesis [8]. Increased ethylene biosynthesis is associated with the final events of abscission activation, namely by promoting CW disassembly-related genes transcription [9, 10]. Increased levels of reactive oxygen species (ROS) have a pivotal role in organ abscission control, encompassing multiple steps of signaling, downstream from ethylene, and associated with ROS-sugar-hormone cross talk [11–14].

In reproductive organs, abscission is also related to lower carbohydrate and polyamine (PA) availability to developing flowers and fruits [15–18]. Together with its role as energy source, glucose acts as a repressing signal of programmed cell death (PCD) [19]. A glucose gradient in the AZ was recently suggested, similar to the auxin flux that regulates ethylene signaling [2]. In addition, the inflorescence deficient in abscission (IDA) peptide signals and interacting receptor-like-kinases, HAESA and HAESA-like2, were showed to activate mitogen-activated protein kinase (MAPK) cascades leading to the abscission of floral organs in *Arabidopsis thaliana* L. [20, 21], in a signaling system that was proposed to be conserved and regulate cell separation in other plant species [22].

Strategies that stimulate flower and fruit abscission are widespread horticultural practices, collectively known as thinning. In seedless table grape (*Vitis vinifera* L.) production, reduction of the number of berries per bunch is mandatory to guarantee bunch quality and decrease fungal diseases incidence [23]. Gibberellic acid (GAc) spraying during bloom, often followed by hand adjustments, is the most common method for thinning in grapevine [23–27], although the mechanisms by which GAc induces abscission remains largely unknown. Gibberellin (GA) perception and signaling investigated in model plants [28] disclosed early recognition *via* the GA INSENSITIVE DWARF1 (GID1) receptor and interaction between GA-GID complex and DELLA transcription factor responsible for GA signaling repression. Binding of GA-GID1 to DELLA induces recognition of DELLA for ubiquitination by a specific F-box protein (GID2) that results in a rapid degradation of DELLAs *via* the ubiquitin-proteasome pathway. Recently, GA-induced changes in the transcriptome of pre-bloom inflorescences and of berry enlargement stages in grapevine were investigated [29, 30] and the results suggested that GAc application to grape flowers and berries has a fairly comprehensive impact on their metabolism mediated by hormone biosynthesis and signaling, in particular through a negative feedback regulation of GAs biosynthesis and signaling [29, 30].

Flower abscission can also be boosted by shading conditions (70-90 % light interception) during bloom [12, 31, 32], paving the way to explore light management as an alternative thinning method. The pronounced reduction of net photosynthetic rates under shading promotes the competition for photoassimilates between vegetative and reproductive organs, leading to shedding of the later with less sink strength at this early stage of development [33]. Shade-induced changes in the transcriptome of apple (*Malus* × *domestica*) revealed that photosynthesis repression and associated nutrient stress is perceived at the fruit level, its growth is inhibited by a sugar transport blockage, resulting in a decreased auxin transport to the AZ and concomitant increased sensitivity to ethylene, leading to fruit abscission [18].

Therefore, abscission is a challenging biological question that can be induced by at least two distinct stimuli with distinct physiological basis. Recently, using an experimental assay with potted seeded vines managed under a greenhouse hydroponic production system, and thinned with GAc spraying or *via* shade nets to reduce intercepted light, we established an efficient method to produce sample sets with predictable abscising potential triggered by different (chemical and environmental) cues, which allowed us to disclose the participation of different metabolic pathways according to the imposed treatment in flower abscission regulation [12]. We now report the effect of the same abscission-inducers using a different genetic background under field conditions. The rationale was that, by using a seedless variety deprived of the main endogenous source of bioactive GAs [34] and developed while adapting to field multiple stresses, the major signals for abscission triggering would be perceived, providing new insights on this subject. Hence, comprehensive cutting-edge metabolomics, RNA-Seq transcriptomics and physiological measurements, were performed to allow discussing how environmental (C-shortage) and GAc application act to trigger flower abscission, to identify routes linking the aptitude of an organ to become competent for cell separation and specificities and communication between different pathways leading to organ drop. In addition, the present study provides the first sequential transcriptomic atlas of GAc-induced flower abscission.

## Methods

### Experimental conditions and sample collection

The trail was conducted in a commercial table grape vineyard in south of Portugal (38° 05' 23.80" N; 8° 04' 52.7 1" W), using seven-year-old ‘Thompson Seedless’ (*Vitis vinifera* L.) vines grafted on ‘140 Ruggeri’ rootstock, spaced 3x3 m, grown under an overhead trellis system covered with plastic, and managed following standard fertilization, irrigation, and pest-management practices. Permission to access and sample at the vineyard was previous agreed under the frame of joint research partnership.

The imposed treatments were: thinning *via* reduction of intercepted light and chemical thinning with GAc, in five vines per treatment. An additional group of five vines remained untreated to be used as control. Shade was imposed at 50 % cap fall (stage 65 of the BBCH scale [35]) by covering the vines with polypropylene shading nets (Hubel, Portugal) that intercept 100 % of the incident photosynthetic photon flux density (PPFD), for a period of fourteen days. Chemical treatment consisted in spraying GAc solution (Berelex with 9 % of GAc, Kenogard) at 10 ppm, 12.5 ppm and 12.5 ppm, applied sequentially at 20 %, 50 % and 100 % cap fall (stages 62, 65 and 69 of the BBCH scale, respectively).

**Fig. 1:**
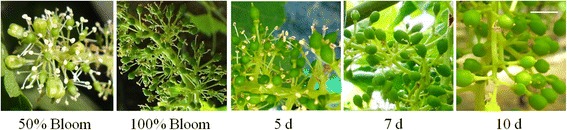
Aspect of 'Thompson Seedless' inflorescences from 50 % cap fall to 10 days after 100 % cap fall. Samples were collected at 5, 7 and 10 days after 100 % cap fall (5, 7 and 10d). Scale bar corresponds to 0.6 cm

Climate conditions during the assay were monitored above the canopy of shaded and control vines (WatchDog MicroStation, Spectrum Tech., USA) (Additional file 1). Grape inflorescence samples were collected in a time-course assay, at three time-points: 5, 7 and 10 days after 100 % cap fall (referred to as 5d, 7d and 10d) (Fig. 1). In each point, three independent biological replicates were collected per treatment in the corresponding five-vine plot. Each biological replicate is composed essentially by the flowers with their pedicels from an inflorescence deprived from rachis, immediately frozen in liquid nitrogen, and subsequently fine-powdered and stored at −80 °C until use.

### RNA deep sequencing and bioinformatic analysis

Total RNA was extracted and purified from ca. 100 mg frozen inflorescences from each 5 and 7d biological sample, using the RNeasy Plant RNA Extraction Kit and RNase-Free DNase Set (Qiagen, Hilden, Germany) following the manufacturer’s instructions, but replacing the extraction solution for a 100 mM Tris-HCl, 2 % (w/v) CTAB, 25 mM EDTA and 2 M NaCl buffer [36]. When traces of contaminant genomic DNA were detected after standard PCR amplification of the *ACTIN* 1 (ACT1) gene (XM_002282480.3), samples were further digested with RNase-free DNase I (Ambion, Life Techonologies, CA, USA). RNA integrity and purity were evaluated by visual inspection of ribosomal bands in 1.5 % agarose gel electrophoresis and by 2100 Bioanalyzer (Agilent Technologies, CA, USA) readings. Poly(A) mRNA isolation, cDNA synthesis, library generation, indexing, cluster generation and RNA-Seq analyses by Illumina HiSeq 2000 RNA sequencing of 100 bp paired-end reads was carried out by LGC Genomics (Berlin, Germany), using commercial services.

The raw Illumina 100-bp pair-end sequences were deposited in the NCBI Sequence Read Archive (SRA). The reads were quality trimmed using Trimmomatic version 0.32 [37], and surveyed for the presence of rRNA contamination using homology searches against rRNA databases [38]. Alignment against the *Vitis vinifera* reference genome [39] was then performed with the software Tophat2 version 2.0.12 [40] set with the parameters -D 15 -R 2 -L 22 -i S,1,1.15 and end-to-end mode. Quantification and normalization of gene expression values by Fragments Per Kilobase Of Exon Per Million Fragments Mapped (FPKM) was calculated by Cufflinks version 2.2.1 [41]. Differential expression calculations were handled by DESeq2 version 1.4.5 [42] considering estimation of size factors, a false discovery rate (FDR) of 0.05 and a -1.5 ≥ log2 fold-change ≥ 1.5, using the raw read counts.

EuKaryotic Orthologous Groups (KOG) [43], Gene Ontology (GO) and Kyoto Encyclopedia of Genes and Genomes (KEGG) [44] functional annotations were based on sequence homologies against public databases. Rapsearch2 [45] with an e-value cut-off of 10^−5^ was used to search against *Arabidopsis thaliana* sequences in the KOG database and non-redundant (“nr”) peptide database (ftp://ftp.ncbi.nlm.nih.gov/blast/db/ downloaded at November 26, 2013, including all “nr” GenBank CDS translations + PDB + SwissProt + PIR + PRF). To GO and KEGG annotations, the output was submitted to an in-house developed script - Rapsearch2XML (https://github.com/Nymeria8/Rapsearch2Xml) and then to Blast2GO [46]. GO enriched categories were identified using the R bioconductor package topGO version 2.18.0 [47], using a Fisher's exact test and a *p*-value ≤ 0.01.

### Data validation by gene expression quantification and correlation between replicates analyses

Aliquots (150 ng) of the same RNA samples extracted as per 2.1 were used for first-strand cDNA synthesis by M-MLV Reverse Transcriptase (Invitrogen), according to the manufacturer’s instructions. The expression of eight genes with significant differences on RNA-seq analysis, involved in auxin and ethylene signaling pathways [9, 48] and mitogen-activared protein kinase cascades [20] putatively related to flower abscission regulation, was assessed by q-rtPCR. Their specific primer sequences and properties are given in Additional file 2. qRT–PCR amplifications were conducted in a qTOWER 2.0 (Analytikjena, Germany) thermal cycler in 15 μL reactions containing 1× SsoAdvanced™ SYBR®Green Supermix (Bio-Rad), 0.3 μM each primer and 90 ng cDNA.

The amplification cycling profile was: 95 °C during 30 s; then 40 cycles at 95 °C for 5 s and 60 °C for 30 s. Melting curves were generated to confirm amplification of single products and absence of primer dimerization. For each primer pair, PCR amplification efficiencies were calculated *via* a calibration dilution curve and slope calculation, using the equation E(%) = (10^[−1/slope]^) × 100 [49]. Data normalization was conducted based on quantification threshold cycle (Ct) values with respect to the geometric average of the Ct of 3 reference genes [50], polyubiquitin (XM_002282083.2), actin (XM_002282480.3) and glyceraldehyde-3-phosphate dehydrogenase (XM_002263109.2). Each analysis was performed in duplicate technical reactions, in each of the three biologic replicates per treatment and condition. To obtain measurements of the correlation between RNA-seq and qRT-PCR data, linear regression and determination coefficient (R^2^) were determined between the two methods obtained log_2_ fold-changes for the same eight genes.

To further investigate the robustness of our RNA-seq dataset, similarity of expression profiles between the three biological replicates was determined by Pearson correlation coefficient (PCC) analyses with R 3.1.2 software using natural logarithm (ln)-transformed read counts for the differentially expressed genes (DEG) as input.

### Global and targeted metabolomic profiling

Circa 200 mg of powdered material from each of the three biological replicates collected at 5d and 7d for each treatment were lyophilized, extracted with methanol and analyzed using the integrated platform developed by Metabolon® (Durham, USA) consisting of a combination of three independent approaches: ultrahigh performance liquid chromatography/tandem mass spectrometry (UHLC/MS/MS2) optimized for basic species, UHLC/MS/MS2 optimized for acidic species, and gas chromatography/mass spectrometry (GC/MS). Methods were performed as previously described [51–53]. For UHPLC/MS/MS2 analysis, aliquots were separated using a Waters Acquity UHPLC (Massachusetts, USA) and analyzed using a LTQ linear ion trap mass spectrometer (Thermo Fisher Scientific Inc., Massachusetts, USA). Each extract was monitored for positive or negative ions in independent injections using separate acid/base dedicated 2.1 mm × 100 mm Waters BEH C18 1.7 μm particle columns, heated to 40 °C. The MS interface capillary was maintained at 350 °C. The spray voltage for the positive ion injection was 4.5 kV, and 3.75 kV for the negative ion injection. The instrument scanned 99-1000 m/z and alternated between MS and MS/MS using dynamic exclusion with approximately 6 scans per second. MS/MS normalized collision energy was set to 40, activation Q 0.25, and activation time 30 ms, with a 3 m/z isolation window. MS/MS scans were collected using dynamic exclusion with an exclusion time of 3.5 s. Derivatized samples for GC/MS were separated on a 5 % phenyldimethyl silicone column with helium as the carrier gas and a temperature ramp from 40 °C to 300 °C and then analyzed on a Thermo-Finnigan Trace DSQ MS (Thermo Fisher Scientific Inc., Massachusetts, USA) operated at unit mass resolving power with electron impact ionization and a 50–750 atomic mass unit scan range.

Metabolites were identified by automated comparison of the ion features in the experimental samples to a reference library of chemical standard entries that included retention time, molecular weight (m/z), preferred adducts, and in-source fragments as well as associated MS/MS2 spectra (Additional file 3) and curated by visual inspection for quality control using a software developed at Metabolon Inc [53]. Raw area counts for each biochemical compound were rescaled by dividing each sample’s value by the median value for the specific biochemical. Welch’s two-sample t-tests were then used to determine whether or not each metabolite had significantly increased or decreased in abundance using Array Studio software (Omicsoft) and Microsoft Excel® spreadsheets. Mapping of metabolites was performed onto general biochemical pathways, as provided in the Kyoto Encyclopedia of Genes and Genomes (KEGG) (www.genome.jp/kegg/) and Plant Metabolic Network (PMN) (www.plantcyc.org/).

Hormone (indole-3-acetic acid (IAA), abscisic acid (ABA), GA_1_, GA_4_, GA_8_, GA_9_, GA_12_, GA_20_, GA_34_, GA_53_) extraction and quantification were performed [54] in 5d, 7d and 10d inflorescence samples. Starting from lyophilized ca. 300 mg weighed aliquots per sample, 15 μL samples were injected on an Acquity UPLC BEH C18 column (1.7 μm film thickness, 2.1 mm × 100 mm; Waters) mounted into an Acquity UPLC Waters equipped with a Xevo TQ MS mass spectrometer (Waters Corporation, Milford, USA). Flow rate was set at 0.45 mLmin^−1^ and column temperature at 40 °C. Eluent A was a 0.1 % formic acid in a 2 mM ammonium acetate solution and eluent B was methanol with 0.1 % formic acid in a 2 mM ammonium acetate solution. Chromatographic separation was obtained using the following gradient for solvent B: 2 % for 0.5 min, raised to 95 % in 7.25 min, then held at 95 % for 1 min, and back to 2 % in 0.01 min. Column reconditioning was performed holding B at 2 % per 3 min before each injection. The transitions are reported in Additional file 4. Sugar (glucose, sucrose, fructose and stachyose) and free PA (putrescine, spermine, spermidine and cadaverine) contents from inflorescence samples collected at same time points were extracted and quantified by high performance liquid chromatography (HPLC) as previous described by [12]. To access the significance of the differences between treatments, one-way ANOVA and Tukey HSD test at *p*-value ≤ 0.05 were performed using Statistix9 software.

### Exploratory analysis of transcriptome and metabolome profile

Data regarding transcript and metabolite quantification was natural logarithm (ln) –transformed for adjustment to normal distribution and verified by histogram plotting, using the R software before and after the transformation. Principal Coordinate Analysis (PCoA) was conducted based on the pair-wise correlation matrix using the NTsys-PC 2.20e software [55]. The DCENTER module was used to transform the symmetric matrix to scalar product and EIGEN for eigenvalues decomposition to identify orthogonal components of the original matrix modules. The minimum-spanning tree was calculated allowing the visualization of the distances between operational units. R software was used for Orthogonal Signal Correction Partial Least Squares Discriminant Analysis (O-PLS-DA) and heatmap construction with associated hierarchical clustering. Approximately unbiased and bootstrap probability *p*-values were calculated using pvclust version 1.3.2 [56] with UPGMA method and 1000 bootstrap replications.

### Vine physiology and final bunch morphology assessment

Flower drop was monitored with resource to non-woven cloth bags positioned around 10 bunches per treatment at full bloom and kept until 10d (days after 100 % cap fall). Shoot length and primary and secondary leaf areas were determined at bloom and 15 after, in six shoots per treatment, following non-destructive methods [57]. Estimated leaf chlorophyll content (SPAD-502 m, Minolta, Japan) was measured twice during the shade period (2 and 9d). Leaf gas exchange were measured in the morning period (9:00 am - 11:00 am) using a portable CO_2_/H_2_O porometer (CIRAS-1, PPSystems, USA), on eight mature leaves from the central part of the shoots, twice during the shade period (8 and 10d) and twice after removal of the shading nets (30 and 43d). At harvest (96d), the same bunches used for flower drop monitoring, were collected and the final number of berries was recorded to calculate the flower drop percentages. Bunch weight, rachis length and bunch compactness (number of berries cm^−1^ of rachis) were also determined. To access the significance of the differences between treatments, one-way ANOVA and Tukey HSD test were performed as previous described for targeted metabolite analysis.

## Results

### Effects of GAc and shade on leaf gas exchanges, vegetative and reproductive organs development

During bloom period, no significant differences on the day/night mean temperature and relative humidity were perceived between treatments which were in average 26/15 °C and 57/71 %, respectively (Additional file 1). Conversely, 100 % PPFD interception was observed in the shaded vines. Leaf net photosynthetic rate (P_n_), stomatal conductance (*g*_*s*_), vegetative growth and chlorophyll content were reduced, only under shaded conditions (Table 1). Increased shoot growth was observed in vines submitted to GAc treatments, when compared with shade-treated vines.

**Table 1:** Effect of GAc and shade treatments on physiological measurements during shade period

	P_n_ (μmol CO_2_ m^−2^ s^−1^)	*g*_*s*_ (mmol H_2_O m^−2^ s^−1^)	Leaf chlorophyll content (SPAD units)	Total leaf area growth (m^2^ vine^−1^ day^−1^)	Shoot growth (cm day^−1^)	Flower drop (%)
Control	8.7 a	83.5 a	25.6 a	0.914 a	2.9 ab	63.1 c
GAc	8.8 a	83.4 a	24.2 a	0.917 a	3.8 a	83.0 b
Shade	0.0 b	7.4 b	22.5 b	0.052 b	1.6 b	99.0 a
	***	***	***	***	**	***

Net photosynthetic rate (P_n_), stomatal conductance (*g*_*s*_), estimated leaf chlorophyll content, total (primary and secondary) leaf area growth, shoot growth and total percentage of flower drop average values are reported. **, *** mean that the treatments are significantly different at *p*-value ≤ 0.01 or ≤0.001 (ANOVA). Within each column, different letters indicate significant differences among treatments according to Tukey’s HSD test (*p*-value ≤ 0.05)

GAc and shade treatments resulted in the drop of 887 ± 74 and 955 ± 9 flowers per inflorescence, respectively, corresponding to 83 % and 99 %. These values were significantly higher as compared to the control (natural drop flower) that showed a loss of 569 ± 81 flowers, corresponding to 63.1 %. Therefore, both GAc and shade imposed treatments significantly induced flower abscission, although with a higher magnitude resulting from light interception, validating our experimental setup. After shade removal, leaf gas exchange rates recovered to values not significantly different from control.

At harvest, the increased flower abscission was translated in a reduced berry number and bunch compactness in both treatments (Table 2). Rachis length and bunch weight and yield were also reduced in bunches from shade-treated plants.

**Table 2:** Effect of treatments on gas exchange rates after shade period and bunch quality at harvest

	Pn (μmol CO_2_ m^−2^ s^−1^)	*g*_*s*_ (mmol H_2_O m^−2^ s^−1^)	Bunch weight (g)	Number of berries	Rachis length (cm)	Bunch compactness
Control	7.3	69.7	1479.6 a	324.2 a	48.5 a	6.8 a
GAc	8.6	81.8	821.8 ab	168.0 b	44.9 a	3.9 b
Shade	7.0	92.6	97.0 b	14.8 c	20.8 b	0.7 c
	ns	ns	**	***	***	***

Net photosynthetic rate (P_n_) and stomatal conductance (*g*_*s*_) after shade period, and bunch weight, number of berries, rachis length and bunch compactness average values are reported. ns, **, *** mean that the treatments are not significantly different, are significantly different at *p*-value ≤ 0.01 or ≤0.001 (ANOVA). Within each column, different letters indicate significant differences among treatments according to Tukey’s HSD test (*p*-value ≤ 0.05)

### Transcriptome analysis

Eighteen RNA-seq 100-bp paired-end read libraries were prepared from poly(A) RNA extracted from grapevine inflorescences and an average of 27 million paired end reads were collected per each library (Table 3). Approximately 8 % of the reads were trimmed based on the presence of Illumina adapters or low quality bases. After removing rRNA contamination, clean reads were obtained and the statistics of each sample mapping are showed in Additional file 5. Reads mapping to the genome sequence made up approximately 76.8 ± 1.8 % of the reads (Table 3).

**Table 3:** RNA-Seq data overview

	Raw read pairs (x1000)	Remaining reads after trimming (%)	Mapped reads (%)
C5d	36342 ± 5193	91.1 ± 2.5	76.9 ± 0.7
C7d	24725 ± 603	92.1 ± 0.8	76.1 ± 0.7
GAc5d	23705 ± 1936	93.2 ± 0.8	77.9 ± 2.2
GAc7d	20957 ± 1580	91.1 ± 1.3	76.0 ± 0.7
SH5d	26103 ± 1920	92.4 ± 1.0	80.0 ± 5.5
SH7d	30549 ± 1242	92.2 ± 1.6	74.1 ± 1.0

Reads number obtained in each treatment, percentage of reads after data trimming and of successfully mapped reads after rRNA contamination removal (mean of three independent biological replicates ± standard error (se))

**Fig. 2:**
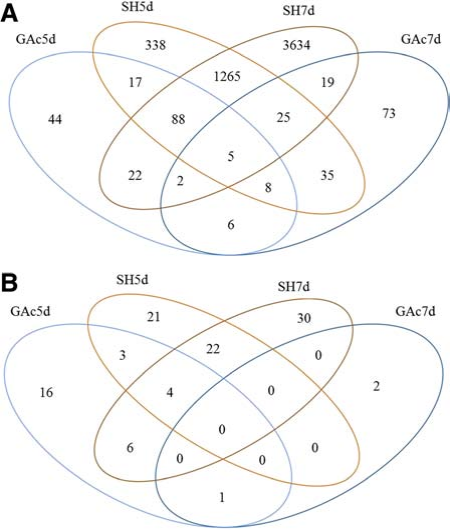
Diagram representing the number of DEG (**a**) and differentially changed metabolites (**b**) in treated inflorescences. Values indicate unigenes passing cut-off values of −1.5 ≥ log2 fold change ≥1.5 and *p*-value ≤ 0.05for transcripts, and *p*-value ≤ 0.05 for metabolites affected by GAc and shade treatments relatively to the control. The list of all DEG, their respective annotation, fold-change and KOG functional category are given in Additional file 6

**Fig. 3:**
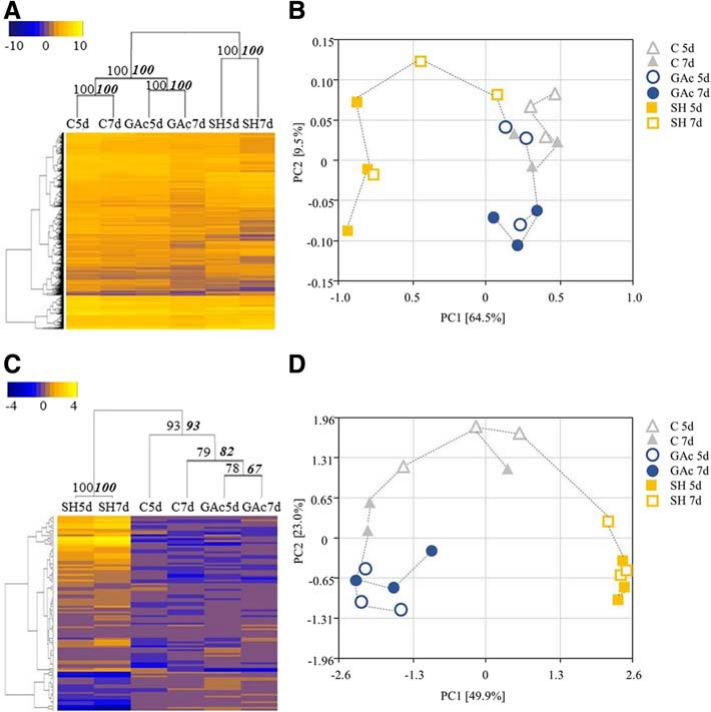
Hierarchical clustering and principal coordinate analysis (PCoA) of transcriptomic and metabolomic profile. Hierarchical clustering of expression values (**a**) and metabolite content (**c**) at different sampled stages. Each column represents the mean value for each treatment at each sampled stage (5 and 7 days after cap fall (**d**)). Data were ln-transformed and yellow tones represent higher values while blue tones represent lower values. The strength of dendrogram nodes was estimated with a bootstrap analysis using 1000 permutations, values represented in the left side of internal nodes are the approximately unbiased *p*-values (AU), bold and italic values on the right side represented the bootstrap probability value. Principal Coordinate Analysis of expression values (**b**) and metabolite content (**d**) of control (triangles), GAc (circles) and shade (squares) treated inflorescences, at 5d (open) and 7d (close), and respective biological replicates. The variance explained by each coordinate (%) is given under brackets

A total of 5581 genes were identified as differentially expressed between control and at least one of the libraries from treated samples (Additional file 6). The abbreviations GAc5d, GAc7d, SH5d and SH7d mean the log2 fold-change between gene relative expression obtained in treated and control inflorescences, from samples collected at 5 and 7 days after 100 % cap fall. As shown in Fig. 2a, the shade treatment was responsible for the highest number of DEG, with 1781 and 5060 genes significantly showing differential expression at 5 and 7d, respectively. On the other hand, GAc treatment led to the differential expression of 192 and 173 genes, in 5d and 7d samples, respectively. According to hierarchical clustering analysis, means of expression values of samples collected in the two time points investigated from each thinning treatment, were significantly clustered together (Fig. 3a). Regarding PCoA, the shade-treated biological replicates were differentiated from GAc and control ones by PC1 in both time points, whereas PC2 separated the 7d GAc-treated biological replicates from the controls (Fig. 3b). These results indicate that, while shade treatment affected significantly the overall transcriptome dynamics both at 5 and 7d, in GAc, only in the second time sampled the treatment effect was above the biological variation between replicates. The OPLS-DA analysis of the differential expressed genes plotted by the KOG categories showed an overlap of gene functional categories (Additional file 7A). A positive significant correlation was found between the log2 fold-changes from qRT-PCR and RNA-Seq transcriptomic datasets, confirming the reproducibility of RNA-Seq data (Additional file 2). In agreement, the robustness of the generated RNA-Seq dataset was further revealed by a high correlation of the transcriptome profiles among three biological replicates per treatment (Additional file 8).

### Metabolome analysis

Regarding global metabolomic analysis, from the 215 metabolites searched by the global metabolic analyses conducted, a total of 105 changed its relative content in at least one of the conditions (*p*-value ≤ 0.05) (Additional file 9). For the Fig. 2b, the abbreviations GAc5d, GAc7d, SH5d and SH7d mean the log2 fold-change between metabolite relative content obtained in treated and control inflorescences, collected after 5 and 7 days after 100 % cap fall. In samples from the GAc treatment, 30 and three metabolites changed respectively at 5 and 7d, while in shaded vines, 50 and 62 metabolites changed in the same time points (Fig. 2b). According to hierarchical clustering, the two time points of each treatment were clustered together and the different treatments were separated with strong confidence based on bootstrap analyses (Fig. 3c). Figure 3d shows the association between biological replicates from all samples. PC1 separated shade from GAc treatment, while PC2 distinguished control replicates from treated ones, in both time points. According to OPLS-DA, metabolites clustered by super-pathway showed specific distribution patterns (Additional file 7B). Altered metabolites derived from amino acid metabolism are identified as the major source of the variance in our data set. The results also show that component 1 clearly separated changes on metabolites from peptide metabolism from secondary metabolites and, to a less extent, from carbohydrates, lipids and nucleotides (Additional file 7B).

### Functional annotation and enrichment analysis

From the total 5581 DEG, 2079 were automatically classified in KOG functional categories, 748 were manually assigned to the same categories according to the similarity with the automatically annotated, 393 were assigned to other functions and 2361 were classified as general or unknown function (Additional file 6). The most representative functional categories in shade-treated samples at both time points investigated were: signal transduction mechanisms, secondary metabolites biosynthesis, transport and catabolism, carbohydrates transport and metabolism, transcription and posttranslational modification, protein turnover, and chaperones (Additional file 10). At the metabolite level, the most representative pathways included amino acid and peptide, carbohydrate, lipid and cofactors metabolism in both time points, whereas secondary metabolism and nucleotide metabolism were most representative only at 5d and 7d, respectively.

To cope with the exploratory analysis results observed at transcriptome level (Fig. 3a), only GAc-treated samples collected at 7d will be discussed. In this sample set, energy production and conversion, translation and ribosomal structure, carbohydrates transport and metabolism, transcription and signal transduction mechanism functional categories were the most representative functional categories (Additional file 10). Based on metabolome analysis, carbohydrates, amino acid and peptide, secondary metabolism, nucleotide and cofactor, prosthetic group and electron carrier metabolism were the most representative superpathways at 5d, while nucleotide, hormone and cofactors metabolisms were the only classes represented at 7d in GAc treated samples.

In addition, enzyme identification among DEG and its KEGG metabolic pathway assignment allowed identifying 24 and 205 enzymatic classes and 32 and 113 KEGG pathways for GAc- and shade-abscission inducing treatments, respectively (Additional file 11). The most representative KEGG metabolic pathways were oxidative phosphorylation and purine metabolism in GAc-treated inflorescences, and starch and sucrose metabolism and purine metabolism in shade-treated inflorescences. According to GO enrichment analysis, which demonstrate if a given pathway is predominant in our data set comparing to whole-genome background (*p*-value ≤ 0.01, Additional file 12), 460 terms were found to be enriched. Acyclic graphs showing the top 5 and top 5-related GO terms mostly affected in treatment and time point (Additional file 13) suggested that genes related to electron and proton transport, oxidative phosphorylation were enriched in GAc-treated inflorescences while genes involved in response to light signal and secondary metabolism were enriched in shade samples, concerning biological processes. Among molecular functions, terms were mostly related to NADH oxidoreductase and dehydrogenase and rRNA binding in GAc-treated inflorescences, and to oxidoreductase, electron carrier, tetrapyrrole binding, hydrolase, glycosyl transferase and phenylalanine ammonia-lyase activities in shade-treated inflorescences. Regarding cellular components, the most enriched categories induced by GAc treatment were intracellular membrane-bounded organelle, chloroplast and cytoplasm, while apoplast, thylakoid and CW terms were enriched in shade treatment.

### Effect of GAc treatment on metabolic pathways

As shown in Table 4, the specific genes most affected by GAc treatment were all up-regulated. The most representative category was energy production and conversion, comprising genes encoding ATP synthases, cytochrome *c* biosgenesis protein, cytochrome oxidase, NADH dehydrogenases, an ATPase, and ribosomal proteins.

**Table 4:** List of top ten DEG specific of GAc treatment

Gene ID	GAc5d	GAc7d	Annotation	UniprotKB	Functional category
VIT_09s0070g00890		1.96	Ribosomal protein S7	F6I3F7	Translation, rib. struct. and biog.
VIT_00s0246g00230		1.98	Cytochrome oxidase subunit III, predicted	F6HML2	Energy product. and conversion
VIT_10s0003g04310		2.00	Vacuolar H + -ATPase V0 sector, subunits c/c'	D7TKE9	Energy product. and conversion
VIT_08s0056g01050		2.03	NADH dehydrogenase subunit 1 (chloroplast)	F6HMW3	Energy product. and conversion
VIT_14s0030g00680		2.05	Ribosomal protein S4, predicted	D7TUX0	Translation, rib. struct. and biog.
VIT_00s0198g00060		2.06	Ribosomal protein S7, predicted	F6I245	Translation, rib. struct. and biog.
VIT_00s0246g00170		2.10	Cytochrome c biogenesis protein (chloroplast)	F6HMK6	Energy product. and conversion
VIT_00s0854g00040	1.50	2.11	NADH dehydrogenase subunit 4 (mitochondrion)	F6HWW5	Energy product. and conversion
VIT_09s0002g00310		2.21	ATP synthase F0 subunit 6, predicted	D7TZJ7	Energy product. and conversion
VIT_14s0036g01270	1.55	2.44	ATP synthase F0 subunit 6, predicted	E0CU73	Energy product. and conversion

Gene code identification, fold-change, annotation, UniProtKB accession number and KOG functional category are showed. Data were obtained from 3 independent biological replicates

**Fig. 4:**
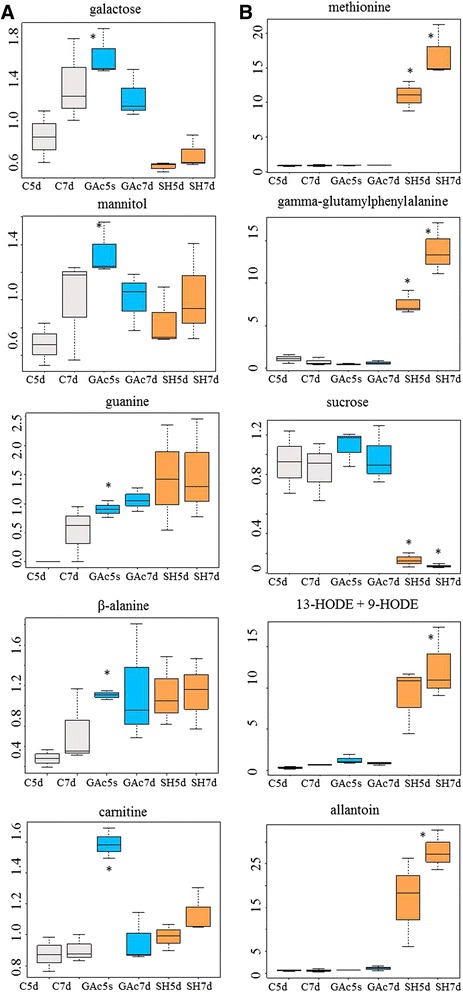
Relative content evolution of the top five metabolites specific of GAc (**a**) and shade (**b**) treatments. Asterisks identify which treatment is different from the control. Data were scale imputed median = 1. Gray, blue, and orange represent samples from control, GAc and shade treatments, respectively. Data were obtained from 3 independent biological replicates

**Fig. 5:**
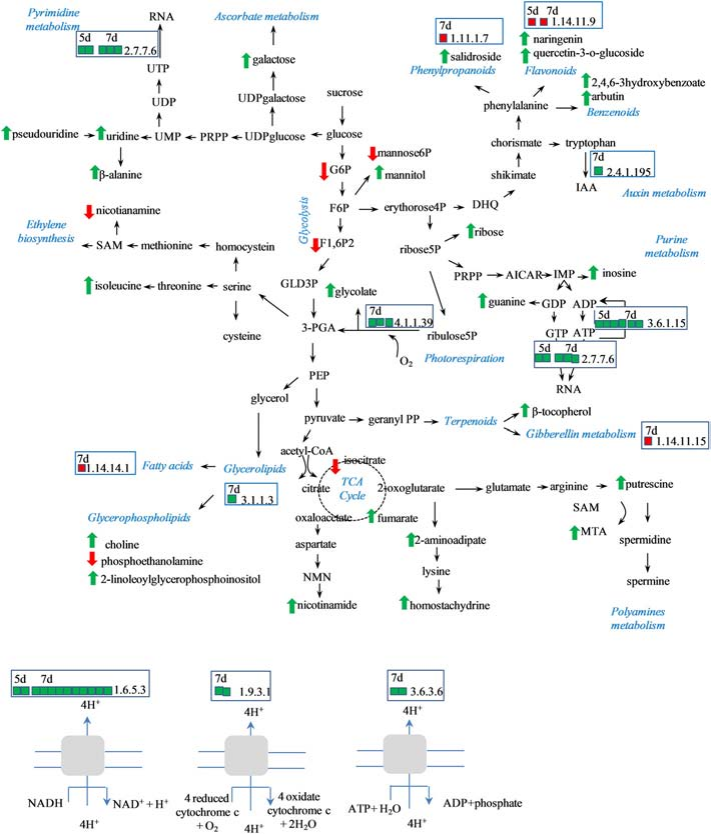
Changes on transcriptomic and metabolic profiles mapped onto simplified metabolic pathways, observed in GAc-treated inflorescences. Red and green squares represent down and up-regulation of the transcripts, respectively. Gene description and fold-change corresponding to enzyme codes are given in Additional file 11. Red and green arrows represent decreased and increased metabolite accumulation, respectively. Description of enzyme codes: 1.11.1.7 - peroxidase; ec:1.14.11.15 - 3beta-dioxygenase; 1.14.11.9 - 3-dioxygenase; 1.14.14.1 - monooxygenase; 1.6.5.3 - reductase (H + -translocating); 1.9.3.1 - cytochrome-c oxidase; 2.4.1.195 - S-beta-glucosyltransferase; 3.1.1.3 - lipase; 3.6.1.15 - nucleoside-triphosphatase; 3.6.3.6 - ATPase; 4.1.1.39 - carboxylase

The most abundant metabolites specifically altered in result of the GAc treatment, were β-alanine and guanine from nucleotide metabolism, carnitine from cofactor metabolism and mannitol and galactose from carbohydrates metabolism (Fig. 4). Giberellate was only detected in GAc treated samples at both time points, presumably of exogenous origin. Targeted metabolite analysis, allowed detecting increased putrescine and GA_8_ molecules and to confirm the rise of GAc in GAc-treated inflorescences at 7d (Table 5). Cadaverine, IAA, GA_1_, GA_4_, GA_9_, GA_12_, GA_20_, GA_34_, GA_53_ readings were below the detection threshold, so could not be quantified. Spermine, spermidine, glucose and fructose contents were not different between treated inflorescences and control. Due to the relatively lower number of GAc-induced alterations particularly when compared to those triggered by shade imposition, it was possibly to map it onto simplified metabolic pathways (Fig. 5).

**Table 5:** Changes on metabolite relative content assessed by target chromatography in treated inflorescence comparing to control

Metabolite	GAc 5d	GAc 7d	GAc 10d	SH 5d	SH 7d	SH 10d	Super pathway
Sucrose				−1.19	−1.60	−1.63	Carbohydrate
Putrescine		0.52		−1.69	−1.40		Polyamines
Abscisic acid				−0.94	−0.71		Hormones
Gibberellic acid		2.36					
Gibberellin 8		1.81					

Metabolite, respective fold-change and super pathway are reported. Data were obtained from 3 independent biological replicates

#### Changes on carbohydrate, cofactor, amino acid and nucleotide metabolism and energy production processes

Glucose-6-phosphate (G6P), fructose-1,6-bisphosphate (F1,6P2) and mannose-6-phosphate (M6P), fructose and mannose levels were reduced, while mannitol, which can be synthesized *via* M6P degradation, and galactose increased in inflorescences from GAc-treated vines. Enhanced photosynthetic and respiratory metabolisms can be hypothesized based on the up-regulation of genes encoding photosystem I and II associated proteins, ribulose-1,5-bisphosphate carboxylase-oxygenase (RuBisCO, EC 4.1.1.39), NADH dehydrogenases (EC 1.6.5.3) and cytochrome-c oxidases (EC 1.9.3.1) and increased glycolate relative content (Additional file 6). Isocitrate relative content decrease and fumarate increase were observed, both associated with the TCA cycle. Cofactors metabolism was also affected, as disclosed by decreased relative contents of nicotianamine and increased nicotinamide and carnitine.

Amino acid and nucleotide pathways were favored in response to GAc treatment comparing to controls, as revealed by increased lysine, isoleucine and polyamine metabolisms and increased pyrimidine and purine metabolisms, respectively (Fig. 5). Conversely N-acetylputrescine levels, involved in putrescine degradation declined. Genes encoding nucleoside-triphosphatase (EC 3.6.1.15), RNA polymerases (EC 2.7.7.6) and H^+^-translocating ATPase (EC 3.6.3.6) were up-regulated.

#### Changes on hormone biosynthesis, transcription factors and lipid and secondary metabolism

A gene encoding an S-beta-glucosyltransferase (EC 2.4.1.195) involved in indole-3-acetic acid (IAA) biosynthesis and secondary metabolism, was up-regulated following GAc treatment.

The down-regulation of a gene encoding a gibberellin 3-beta-dioxygenase (GA3ox) (EC 1.14.11.15) was disclosed and *ETHYLENE-RESPONSIVE TRANSCRIPTION FACTOR RAP2-3* (*ERF RAP2-3*) was the only transcript of hormone signaling pathways affected by GAc (Table 11). The expression of a gene encoding a thioredoxin peroxidase (EC 1.11.1.15) and the relative content of β-tocopherol, associated to reactive oxygen species (ROS) detoxification mechanism were also affected.

As showed in Fig. 5, among lipid-related pathways, glycerolipid and glycerophospholipid metabolism, fatty acid degradation and linoleic acid metabolism were represented.

Secondary metabolic pathways were also significantly altered with the increase of salidroside, naringenin and quercetion-3-O-glucoside, 2,4,6-trihydroxybenzoate and arbutin contents and down-regulation of genes encoding a peroxidase (EC 1.11.1.7) and a hyoscyamine 6-dioxygenase (EC 1.14.11.9), acting in phenylpropanoids, flavonoids and benzenoids biosynthesis and metabolism pathways. Two genes from MYB transcription factors family were down-regulated (Additional file 6).

### Effect of shade treatment on metabolic pathways

Shade imposition resulted in a more pronounced change in the number of genes differentially transcribed and metabolites differentially accumulated than GAc spraying (Tables 4 and 6, Fig. 4). As shown in Table 6, secondary metabolism-related genes encoding a specific MYB transcription factor, flavonol synthase and chalcone synthase, genes encoding a cullin protein, a sugar transporter, stem-specific proteins and a small GTPase protein were the most significantly induced genes, specific for the shade treatment.

**Table 6:** List of top ten DEG specific of shade treatment

Gene ID	SH5d	SH7d	Annotation	UniprotKB	Functional category
VIT_11s0016g01320		−6.51	Transcription factor MYB, predicted	F6HGP6	Transcription
VIT_18s0001g03470	−3.26	−5.70	Flavonol synthase, predicted	F6H0T8	Secondary metab. bios. transp. cat.
VIT_04s0043g00650		−5.63	Cullin-1 isoform 1, predicted		Cell cycle control, cell div., chrom. part.
VIT_14s0068g00930	−2.50	−5.32	Chalcone synthase		Secondary metab. bios. transp. cat
VIT_18s0001g11010		5.42	Ca^2+^independent phospholipase A2, predicted	F6H017	Lipid transport and metabolism
VIT_13s0019g03070	3.31	5.46	Small heat-shock protein Hsp26, predicted	F6HNN6	Posttranslational mod., protein turn., chap.
VIT_05s0020g02170	3.99	5.73	Sugar transporter ERD6-like 16-like, predicted	F6HDJ1	Carbohydrate transport and metabolism
VIT_00s0561g00020	3.86	5.73	Stem-specific protein TSJT1-like	D7TYY3	Other
VIT_02s0033g00830		5.75	GTPase Rab11/YPT3, predicted	F6I079	Intracellular traff., secretion, vesic. transp.
VIT_00s0586g00030	3.91	5.80	Stem-specific protein TSJT1-like , predicted	D7UE87	Other

Gene code identification, fold-change, annotation, UniProtKB accession number and KOG functional category. Data were obtained from 3 independent biological replicates

The most affected metabolites, specifically as result of the shade treatment (Fig. 4b) derived from amino acid and peptide (methionine and gamma-glutamylphenylalanine), carbohydrate (sucrose), lipid (13-HODE + 9HODE) and nucleotide (allantoin) metabolisms. Targeted metabolite analysis confirmed the reduction of putrescine and sucrose contents detected in global metabolomic analysis, and provided additional data of a significant decrease of ABA levels 5 and 7d in inflorescences sampled from shade treated plants.

#### Changes on amino acid, peptide and nucleotide metabolism

Amino acids metabolism was largely affected by shade treatment at the transcriptomic level, inducing alterations in phenylalanine, cysteine, methione, glycine, serine and threonine-related pathways, followed by alanine, aspartate, arginine, glutamate, glutamine, tyrosine, tryptophan, valine, leucine, isoleucine, proline and polyamine related paths. This result also observed regarding changes in metabolite accumulation, which encompass increased abundance of 30 amino acids or amino acid-related metabolites and reduced shikimate, putrescine and 4-acetamidobutanoate relative contents in shaded inflorescences. Glutathione and γ-glutamil peptides accumulation was likewise favored in shade treatment (Additional file 6 and Additional file 9).

DEG associated with purine and pyrimidine nucleotides metabolisms were predominantly up-regulated in result of the shade treatment and the same pattern was observed in associated metabolites, except for guanosine and inosine abundance.

#### Changes on carbohydrate metabolism, transport and signaling pathways

Carbohydrate-related pathways were mostly repressed in shaded inflorescences, including photosynthesis, chlorophyll metabolism, carbon fixation, glycolysis, pyruvate metabolism, TCA cycle, starch and sucrose metabolism, pentose phosphate pathway, fructose and mannose metabolism, amino sugar and nucleotide sugar metabolism, galactose metabolism, pentose and glucuronate interconversions and inositol phosphate metabolisms. At the metabolomic level, malate, citromalate, 2-ketogulonate, gluconate, xylose, inositol, glucose and sucrose decreased while fumarate, arabonate and xylonate were showed to increase in samples from the shade treatment.

Alterations on sugar signaling pathways and transport were induced by shade treatment during bloom, as displayed in Table 7. Genes encoding sugar metabolizing enzymes such as threalose-6-phosphate synthases, sucrose synthases and invertases showed a global up-regulation pattern. Genes encoding glucose-6-phosphate translocators and sugar transporter SWEET1 and 3 were predominantly down-regulated, whereas genes encoding sugar transporter SWEET2 and 10, putative hexose transporter and sugar transporters ERD6-like, implicated in transport of sugars out of the vacuole in C-starvation conditions [58] were up-regulated.

**Table 7:**
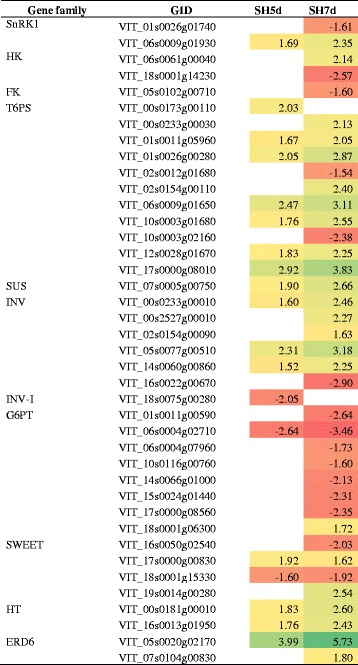
DEG involved in sugar signaling and transport in shade-treated inflorescences and respective fold-change

SnRK1: serine/threonine-protein kinase SnRK1; HK: hexokinase; FK: fructokinse; T6PS: trehalose-6-phosphate synthase; SUS: sucrose synthase; ÍNV: invertase; INV-I: invertase inhibitor; G6PT: glucose-6-phosphate/phosphate translocator 2; SWEET: bidirectional sugar transporter SWEET; HT: hexose transporter; EDR6: sugar transporter ERD6-like

Up-regulation is marked as green and down-regulation as red background. Data were obtained from 3 independent biological replicates

#### Changes on hormone metabolism and signaling pathways

In what concerns hormone metabolism and signaling pathways, genes involved in ethylene and auxin related pathways were highly represented in samples from the thinning by shade treatment (Table 8). Genes encoding S-adenosylmethionine synthase (SAM-S) were down-regulated while the expression of genes encoding ACC oxidases, *ETHYLENE INSENSITIVE 3-LIKE* (*EIN3*) and *ERF*s showed predominantly an up-regulation. Auxin biosynthetic pathway from thyptophan was favored as suggested by the up-regulation of a tryptophan aminotransferase-related gene. Genes encoding auxin binding proteins (ABP) and transport inhibitor response 1 (TIR1) auxin receptors were up-regulated, while *Aux/IAA*, *AUXIN RESPONSIVE FACTOR* (*ARF*) and *AUXIN EFFLUX CARRIERS* (*AEC*) were down-regulated. The synthesis of indole-3-acetic acid (IAA)-amino acid conjugates was induced by the up-regulation of *GH3.9* gene at 5d.

**Table 8:**
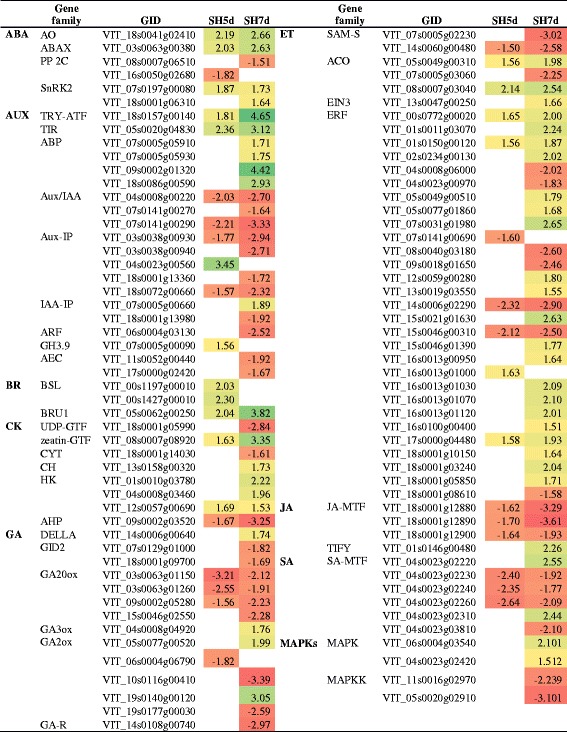
DEG involved in hormone biosynthesis, metabolism and signaling pathways in shade-treated inflorescences and respective fold-change

**ABA:** abscisic acid; AO: aldehyde oxidase; ABAX: abscisic acid 8'-hydroxylase; PP 2C: protein phosphatase 2C; SnRK2: serine/threonine-protein kinase SnRK2; **AUX:** auxin; TRY-ATF: tryptophan aminotransferase-related protein; TIR1 : transport inhibitor response 1; ABP: auxin-binding protein; Aux/IAA: Aux/IAA proteins; Aux-IP: other auxin induced proteins; IAA-IP: other IAA induced proteins; GH3.9: putative indole-3-acetic acid-amido synthetase GH3.9; AEC: auxin efflux carrier component; **BR:** brassinosteroid; BRU1: brassinosteroid-regulated protein BRU1; BSL: serine/threonine-protein phosphatase BSL3-like; **CK:** cytokinin, UDP-GTF: UDP-glycosyltransferase 85A1; ZEA-GTF: zeatin O-glucosyltransferase; CYT: cytokinin riboside 5'-monophosphate phosphoribohydrolase; CYH: cytokinin dehydrogenase; HK: histidine kinase; AHP: histidine-containing phosphotransfer protein; **GA:** gibberellin; DELLA: DELLA protein GAI1; GID2: F-box protein GID2; GA20ox: gibberellin 20 oxidase; GA3ox: gibberellin 3-beta-dioxygenase; GA2ox: gibberellin 2-beta-dioxygenase; GA-R: gibberellin-regulated protein; **ET:** ethylene, SAM-S: S-adenosylmethionine synthase; ACO: 1-aminocyclopropane-1-carboxylate oxidase; EIN3: ethylene insensitive 3-like; ERF: ethylene-responsive transcription factor; **JA:** jasmonic acid; JA-MTF: jasmonate O-methyltransferase; TIFY: TIFY 9 protein; **SA:** salicylic acid; SA-MTF: salicylate O-methyltransferase; MAPKs: mitogen-activated protein kinase cascade: MAPK: mitogen-activated protein kinase; MAPKK: mitogen-activated protein kinase kinase

Up-regulation is marked as green and down-regulation as red. Data were obtained from 3 independent biological replicates

The expression of genes encoding gibberellin20-oxidase (GA20ox), gibberellin3-beta-dioxygenase (GA3ox) and gibberellin2-oxidase (GA2ox) was also significantly regulated (Table 8). GA signaling pathway was repressed, with a concomitant up-regulation of a *DELLA* gene and down-regulation of *GID2*, responsible for DELLA degradation [59].

Genes involved in CK activation, such as those encoding a UDP-glycosyltransferase 85A1 (EC 2.4.1.215), zeatin O-glucosyltransferase and CK riboside 5'-monophosphate phosphoribohydrolase were significantly affected by the imposition of the shade treatment. Genes encoding the CK receptors histidine kinases and histidine-containing phosphotransferase, and CK dehydrogenase enzyme, involved in its degradation, were induced. Shade also promoted the up-regulation of genes involved in brassinosteroids (BR) signal transduction. In addition, the expression of genes encoding cyclin-D3 (CYCD3) proteins, which are downstream components of the CK and BR-signaling pathways that promotes cell division [60], was significantly down-regulated, and a *SENESCENCE RELATED GENE* (*SRG1*) was up-regulated, in inflorescences from shaded vines, at 7d (Additional file 6).

Genes encoding ABA synthesis and degradation enzymes, such as aldehyde oxidase and abscisic acid 8'-hydroxylase, respectively, were up-regulated. These changes on ABA metabolism were also verified as decreased ABA relative content in shaded inflorescences (Table 4). In the ABA-signal transduction pathway, down-regulation of protein phosphatase 2C, which is a negative regulator of ABA response and up-regulation of SnRK2 were observed, suggesting a de-repression of ABA signaling in shaded inflorescences.

The expression of genes encoding methyltransferase enzymes responsible for conversion of jasmonic (JA) and salicylic acids (SA) in methyljasmonate and methylsalicylate, respectively, was down-regulated. JA-mediated signaling pathway was also affected, as revealed by the up-regulation of a gene encoding TIFY9 which negatively regulates a key transcriptional activator of jasmonate responses [61].

#### Changes on lipid, cofactor and secondary metabolism

Impact on lipid-related pathways was disclosed as glycerolipid, glycerophospholipid and sphingolipid metabolism, fatty acid biosynthesis, elongation and degradation, linoleic and arachidonic acid metabolism, unsaturated fatty acids biosynthesis, alkaloid biosynthesis, ether lipid metabolism and cutin, suberine and wax biosynthesis were affected in shade-treated inflorescences (Additional file 11). In particular, genes encoding lipoxygenase (EC 1.13.11.12) and lipase (EC 3.1.1.3) enzymes were highly represented and mostly up-regulated. At the metabolite level, a global increase of fatty acids, oxylipins (HODE), glycerolipids, sterols and glycerophospholipids was also verified (Additional file 9).

Cofactors metabolism-related pathways were also significantly altered, of which thiamine metabolism was the most represented pathway, followed by vitamin B6 metabolism, riboflavin metabolism, pantothenate and CoA biosynthesis and nicotinate and nicotinamide metabolism at transcriptome level. Phosphate and methylphosphate metabolite levels increased in shade-treated inflorescences.

Secondary metabolic pathways such as phenylpropanoid, stilbenoid, monoterpenoid, diterpenoids, carotenoids, benzenoids, flavonoids and anthocyanin biosynthesis and degradation and cytochrome P450-related pathways were significantly affected during shade in both time points. DEG encoding phenylalanine ammonia-lyases (PALs) (EC 4.3.1.25) which catalyse the first step of phenylpropanoids biosynthetic pathway, and stilbene synthases (EC 2.3.1.95) were predominantly up-regulated. Genes encoding myrcene synthases (EC 4.2.3.20) were up-regulated while (3S)-linalool/(E)-nerolidol /(E,E)-geranyl linalool synthases (EC 4.2.3.25) were down-regulated. Flavonoids and diterpenoids biosynthetic pathways were, conversely, repressed. At the metabolomic level, oleanolate from terpenoids metabolism, ferulate from phenylpropanoid metabolism, and both α- and γ-tocopherols increased, while arbutrin (benzenoid) and salidroside (phenylpropanoid) were reduced in result of shaded inflorescences.

Shade altered the accumulation of non-enzymatic markers of oxidative stress, including increased reduced glutathione (GSH) relative content and decreased ascorbate-related metabolites (Additional file 9). The expression of genes encoding enzymatic antioxidants comprising superoxide dismutase, ascorbate oxidase, ascorbate peroxidase, glutathione peroxidase, peroxiredoxin, thioredoxin, glutaredoxin and glutathione S-transferase was also significantly affected (Table 9). In addition, genes encoding laccase (EC 1.10.3.3), involved in ascorbate metabolism and lignin biosynthesis, were exclusively down-regulated in shade treatment.

**Table 9:**
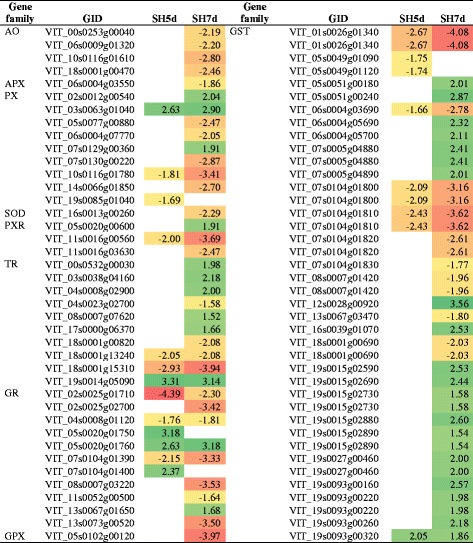
DEG encoding oxidative stress-related enzymes in shade-treated inflorescences and respective fold-change

AO: ascorbate oxidase; APX: ascorbate peroxidase; PX: peroxidase; SOD: superoxide dismutase; PXR: peroxiredoxin; TR: thioredoxin; GR: glutaredoxin; GPX: glutathione peroxidase; GST: glutathione S-transferase

Up-regulation is marked green and down-regulation is marked red

#### Shade-responsive transcription factors

A high number of differentially expressed transcription factors induced by shade treatment was identified, predominantly at 7d, including MYB, GATA, MADS-box, HEX, GT-2, WRKY, CCAAT, ZF-HD, HSF, WOX, E2F/DP, bHLH, MOT2, MEIS1, RF2b and ZFF (Table 10). In particular, genes encoding MYB and GATA families were the most represented and were predominantly down- and up-regulated, respectively. Transcription factors directly involved in hormone signal transduction pathways were represented in Table 8.

**Table 10:**
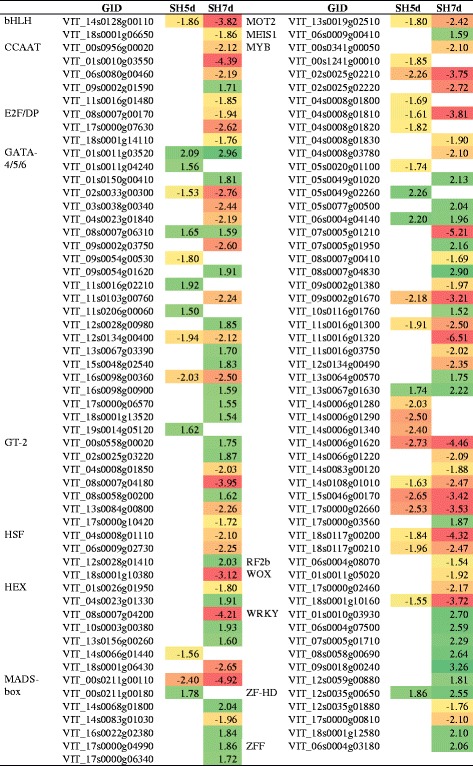
DEG encoding transcription factors in shade-treated inflorescences and respective fold-change

Up-regulation is marked by green and down-regulation by red

### Common DEG and metabolites that significantly changed in response to GAc and shade

In addition to the DEG found to be specific for each treatment, 36 annotated genes were differentially expressed in both abscission inducing treatments, from which 5 DEG changed with a opposite expression pattern, whereas 31 changed followed the same trend (Table 11). The latter ones could be candidate genes involved in shared pathways leading to abscission and were predominantly up-regulated in response to both stimuli. Only five out of these genes, encoding a cooper transporter, subtilisin-like protease, cytochrome P450, a subunit of exocyst complex, and MYB transcription factor, were down-regulated. Genes that showed an opposite change in expression pattern encode a UGT74B1, glucose-6-phosphate translocator, blue Cu-protein and were up-regulated in GAc treatment and down-regulated in shade. Additionally, a gene encoding an ethylene-responsive transcription factor was up-regulated in shade while was repressed in samples from the GAc-treatment.

**Table 11:** List of DEG simultaneous affected by GAc and shade treatments

Gene ID	GAc5d	GAc7d	SH5d	SH7d	Annotation	UniprotKB	NCBI	Functional category
VIT_14s0066g01960	1.71	1.77		3.51	metalloendoproteinase 1, predicted	F6HV36		Amino acid transport and metabolism
VIT_14s0060g00740		1.81		1.80	glycosyl transferase, family 8 - glycogenin, predicted	D7UA70		Carbohydrate transport and metabolism
VIT_07s0129g00790		2.02	1.77		ribulose-1,5-bisphosphate carboxylase /oxygenase subunit	F6HSX2		
**VIT_01s0026g00630**		**1.65**	**−1.99**	**−2.71**	**UDP-glycosyltransferase 74B1, predicted**	**F6HPK7**	**XM_002267629.2**	
**VIT_06s0004g02710**		**1.60**	**−2.64**	**−3.46**	**glucose-6-phosphate/phosphate antiporter, predicted**	**D7SKZ8**	**XM_002285193.2**	
VIT_15s0046g01600		2.11		2.77	acidic endochitinase, predicted	F6I685	XM_002279522.2	Cell wall/membrane/envelope biogenesis
**VIT_18s0001g06580**		**1.60**	**−3.04**	**−3.11**	**blue copper protein-like, predicted**	**F6H0Y2**	**XM_002285700.3**	Coenzyme transport and metabolism
VIT_00s0733g00010		1.83	2.60		ATPase subunit 1 (mitochondrion)	F6I2F8		Energy production and conversion
VIT_00s0332g00170		1.78	2.04		NADH:ubiquinone oxidoreductase, NDUFS2/49 k subunit (mitochondrion), predicted	F6HSD9		
VIT_08s0056g01060		2.25	2.29		NADH dehydrogenase subunit 1 (chloroplast)	F6HMW4		
VIT_14s0108g01640	1.64	2.27	2.56		NADH-plastoquinone oxidoreductase subunit 2 (chloroplast)	F6H5N4		
VIT_13s0067g03310		2.15	1.67		ATPase subunit, predicted	F6HC65		
VIT_01s0011g04110		1.75	2.22	2.39	NADH dehydrogenase subunits 2, 5, predicted	F6HEV2		
VIT_00s0246g00050		1.93	2.32	2.17	NADH dehydrogenase subunits 2, 5, predicted	D7TKT1		
VIT_00s0332g00140		1.61	1.70		NADH:ubiquinone oxidoreductase, NDUFS2/49 kDa subunit, predicted	D7TSH3		
VIT_00s0246g00070		1.96		2.22	NADH:ubiquinone oxidoreductase, NDUFS2/49 kDa subunit, predicted	F6HMJ6		
VIT_10s0116g00060		2.04	2.18		NADH:ubiquinone oxidoreductase, NDUFS2/49 kDa subunit, predicted	E0CVJ6		
VIT_10s0092g00770	1.68	2.27	2.79		cytochrome c biogenesis C (mitochondrion)	F6I3K0	XM_010657573.1	
VIT_03s0110g00360		−2.04		−1.88	copper transporter 6-like, predicted	A5AQX0	XM_003631673.2	Inorganic ion transport and metabolism
VIT_18s0072g00740		−1.55	−1.68	−1.75	sec5 subunit of exocyst complex, predicted	F6GY22		Intracellular traff., secretion, vesicular trans.
VIT_07s0031g00570		1.66		3.41	lipase, predicted	D7SVX1		Lipid transport and metabolism
VIT_18s0001g10330		−1.60		−4.02	subtilisin-like protease	F6H1C2		Posttranslational mod., protein turn., chap.
VIT_00s0332g00010		1.81	2.13		mitochondrial mRNA maturase	F6HSC8		RNA processing and modification
VIT_00s0332g00030	1.57	1.83	2.23		mitochondrial mRNA maturase, predicted	F6HSD0		
**VIT_08s0007g01910**		**1.90**	**−3.05**	**−4.22**	**laccase-4, predicted**	**D7THA7**	**XM_002278602.3**	Secondary metabolites biosynthesis, transport and catabolism
VIT_17s0000g01490		−1.81	−1.88	−2.05	cytochrome P450 94A1-like, predicted	F6GST7	XM_002279945.2	
VIT_02s0012g00820		2.60	2.08		serine/threonine protein kinase, predicted	F6HTC0		Signal transduction mechanisms
VIT_02s0012g00720		2.65	2.14		serine/threonine protein kinase, predicted	D7TTF6		
VIT_12s0028g02570		1.80	2.60	3.46	calmodulin and related proteins (EF-Hand superfamily), predicted	E0CTM8	XM_002279084.2	
VIT_08s0056g00900	1.76	1.87	2.03	2.12	RNA polymerase II, second largest subunit, predicted	F6HMV9		Transcripton
VIT_14s0108g01010	−1.50	−1.90	−1.63	−2.47	transcription factor, Myb superfamily, predicted	F6H5U7		
VIT_09s0002g04540	1.81	2.02	1.96		DNA-directed RNA polymerase subunit beta	D7U0K0		
VIT_15s0024g01960		2.39	2.28		RNA polymerase III, large subunit, predicted	D7UBC3		
**VIT_05s0077g01860**		**−1.56**		**1.68**	**ethylene-responsive transcription factor RAP2-3, predicted**	**D7SYA3**	**XM_002272390.2**	
VIT_00s0173g00170		2.10	1.74	2.33	30S ribosomal protein S7, chloroplastic, predicted	F6HD03		Translation
VIT_10s0092g00790		2.04	2.42		ribosomal protein S19, mitochondrial-like, predicted	D7U8H6		
VIT_02s0033g00990		2.55	2.71		ribosomal protein S7 (chloroplast)	F6I086		
VIT_16s0039g00380		1.77	1.85		mitochondrial/chloroplast ribosomal protein S14/S29, predicted	F6GZ45		
VIT_12s0028g00970		2.20	1.80	2.33	ribosomal protein S7, predicted	F6HRD9		
VIT_09s0070g00900		1.87	1.92	2.37	ribosomal protein S7, predicted	D7U8C4		
VIT_09s0070g00920		1.79	1.84	2.41	ribosomal protein S7, predicted	D7U8C4		
VIT_13s0047g00220		2.12	2.60		mitochondrial/chloroplast ribosomal protein S19, predicted	D7TF26		
VIT_02s0033g00980		2.79	2.88		ribosomal protein S7, predicted	F6I085		
VIT_00s0396g00050		1.81	2.67		ribosomal protein S4 (mitochondrion)	F6HRU1	XM_010648649.1	
VIT_05s0020g00600		1.66		1.91	1-cys peroxiredoxin	D7T674	NM_001281268.1	Other

Gene code identification, fold-change, annotation, UniProtKB accession number and KOG functional category are reported. Data were obtained from 3 independent biological replicates. Bold letters indicate the metabolites showing opposite trend in both treatments

Among the 13 commonly altered metabolites in response to both thinning strategies, eight showed the same pattern in both imposed treatments, belonging mostly to the amino acids pathway (Table 12).

**Table 12:** List of metabolites simultaneous affected by GAc and shade treatments

Metabolite	GAc5d	GAc7d	SH5d	SH7d	KEGG	Pathway	Super pathway
2-aminoadipate	0.52			1.09	C00956	Aspartate family	Amino acid and peptide
N-acetylputrescine	−0.25		−0.92	−1.39	C02714	Glutamate family	
Homostachydrine	0.71		1.39	0.84	C08283		
Isoleucine	0.49		2.26	1.83	C00407	Branched Chain Amino Acids	
5-methylthioadenosine (MTA)	1.17		1.42		C00170	Amine	
Fumarate	0.57			0.46	C00122	TCA cycle	Carbohydrate
**Phosphoethanolamine**	**−0.39**			**1.52**	**C00346**	Phospholipids	Lipid
2-linoleoylglycerophosphoinositol	0.91		0.98			Phospholipids	
**Nicotianamine**	**−1.04**		**1.49**	**1.70**	**C05324**	Nicotinamide	Cofactor
**Inosine**	**0.80**			**−0.55**	**C00294**	Purine	Nucleotide
Pseudouridine	0.85			0.82	C02067	Pyrimidine	
**Arbutine**	**0.51**		**−0.72**		**C06186**	Benzenoids	Secondary metabolism
**Salidroside**	**2.02**		**−2.69**			Phenylpropanoids	

Metabolites, fold-change, KEGG compound number and pathway are reported. Data were obtained from 3 independent biological replicates. Bold letters indicate the metabolites with opposite trend

On other hand, the phospholipid phosphoethanolamine and nicotianamine decreased in GAc treated samples and increased in those from shaded vines, while putrescine, inosine, arbutine and salidorise were increased in GAc- and reduced in shade-treated inflorescences (Tables 5 and 12). Other gene family, vacuolar H^+^-ATPase, was affected by GAc and shade treatments, although not exactly the same genes were involved (Additional file 6).

## Discussion

### What makes a flower to abscise?

Flower abscission depicted by –OMIC approaches disclosed a complex regulation including adjustments of metabolism, gene expression and physiology. In grapevine, natural flower drop occurs between 6 and 12 days after 100 % cap fall (d) [62] and peaks at 10 d under our experimental conditions (data not shown). Our data revealed that GAc and shade induced flower abscission by opposite effects on cell metabolism at 5 and 7 d, but converging on some common pathways leading to abscission.

As previously reported, polyamine metabolism pathway have a key role in reproductive organs abscission [12, 15, 16, 63, 64]. Changed putrescine inflorescence content varied with the imposed treatment, increasing and decreasing in result of GAc- and shade-treatment, respectively. Whereas putrescine catabolism, by conversion on N-acetylputrescine and/or biosynthesis of downstream polyamines spermidine and spermine with the accumulation of 5-methylthioadenosine (MTA), was affected in the same direction in both treatments (Table 12). MTA is produced from S-adenosylmethionine (SAM) through the spermidine and spermine biosynthetic pathway, where it behaves as a powerful inhibitory product [65], suggesting that the regulation of the downstream polyamines biosynthetic step, but not the biosynthesis of its precursor putrescine, is a common signal of abscission. In addition, in inflorescences developing under shaded conditions, the observed up-regulation of a gene encoding SAM decarboxylase (EC 4.1.1.50) and repression of the subsequent step of spermidine biosynthesis, by the down-regulation of a putative *SPERMIDINE SYNTHASE* 2 gene (VIT_17s0000g08030) indicates that this step of polyamines metabolism was also regulated at transcriptome level (Additional file 6). This is in accordance with observations of abscission inhibition by application of exogenous spermidine, but not of putrescine, prior to flowering [15]. MTA is also produced *via* ethylene biosynthetic pathway [66] which was significantly affected only by shade treatment (Table 8), while the expression of *ERF RAP2-3* was induced by shade and repressed by GAc treatment, thus suggesting that the ethylene signal transduction pathway was differentially regulated according to the treatment (Table 11).

Two common events were the up-regulation of both genes involved in RNA metabolism, such as those encoding RNA polymerases and ribosomal proteins, and energy production related genes, such as NADH dehydrogenases, cytochrome *c* and ATPase (Table 11), suggesting an increased demand for energy. NADH:ubiquinone oxidoreductase from NADH dehydrogenase family and cytochrome *c* are members of the respiratory chain, acting to generate a proton gradient which is thereafter used for ATP synthesis through H^+^-transporting ATPase. The up-regulation of chloroplastic NADH dehydrogenases suggested that chlororespiration, which is associated with ROS alleviation around photosystems [67], is also induced as response to both treatments.

In addition to genes encoding serine/threonine protein kinases and calmodulin protein, which are components of signal transduction pathways, a gene encoding a subtilisin-like protease, described to be involved in protein turnover, generation and processing of peptide signals and programmed cell death [68–70], was commonly affected by abscission-inducing stimulus (Table 11). The higher transcript accumulation of a gene encoding a specific antioxidant 1-cys peroxiredoxin enzyme (EC 1.11.1.15), which is prone to be reduced by ascorbic acid or glutathione, was additionally found to be common after both abscising inducing treatments. This observation agrees with previous works that described the multiple ROS roles in abscission including signaling, ROS-sugar-hormone cross talk and induction of the expression of CW-degrading enzymes [11, 14]. Other changes on enzymatic and non-enzymatic oxygen stress remediation mechanisms were found to be specific from each abscission-triggering *stimulus*. In particular, the accumulation of the antioxidants arbutin, salidroside, and the expression of genes encoding a laccase 4 and other blue Cu-protein were contrasting between the two treatments, indicating different ROS detoxification instruments triggered by GAc or by shade treated inflorescences (Tables 11 and 12).

Regarding amino acid metabolism, the observed induction of lysine and isoleucine biosynthetic pathways revealed that both treatments are abiotic stress-impacted. Lysine is a precursor for glutamate, an important signaling amino acid that regulates plant growth and plant-environment responses [71]. On the other hand, isoleucine is accumulated as a compatible osmolyte, playing a role in plant stress tolerance [72]. In lipid-related pathways, changes in glycerolipids and phospholipids metabolism indicated alterations on cell membrane stability and signaling lipids content [73, 74], as candidates to common markers of abscission.

The common event of increased transcription of genes encoding glycogenin and RuBisCO enzymes, suggests that, in what concerns carbohydrate metabolism, conversion of glucose to the energy storage polymer glycogen and CO_2_/O_2_ fixation were affected in both samples (Table 11). At the metabolite level, the accumulation of fumarate (Table 12) was also reported to be associated to flower shedding in response to the same treatments, under greenhouse conditions [12]. Among the multiple functions of fumarate, are the involvement in pH regulation, stomatal movement and signaling and as a respiratory substrate during carbon starvation [75, 76]. Expression of genes encoding vacuolar H^+^-ATPase genes involved in pH regulation was affected by both treatments, although exclusive up-regulation was only found in GAc treated inflorescences (Additional file 6). This overexpression agrees with the recent findings of cytosolic alkalization as part of abscission pathways and occurring concomitantly with the execution of organ abscission [77].

Pathogenesis-related genes, as the *ACIDIC ENDOCHITINASE* up-regulation in inflorescences submitted to both treatments (Table 11), are reportedly expressed at the site where organs will be shed during abscission [78, 79], and proposed to act in establishing a defense system at the plant’s side.

### The GAc abscission inducing mechanism requires energy production and global metabolism stimulation

Although the GAc effect is known to be largely dependent on the microclimate conditions [12], GAc application at bloom was a successful treatment to promote flower abscission (Table 1), and cluster loosening at harvest (Table 2), in 'Thompson Seedless' vines growing in open field conditions.

Considering the higher magnitude of changes on metabolite content observed at 5d comparing to 7d (Additional file 9), together with the most significant transcriptomic reprogramming noticed only at 7d (Fig. 3b), one may hypothesize that the process by which exogenous application of GAc significantly increased flower abscission, could result of non-enzymatic mechanisms. Non-enzymatic reactions are an integral part of metabolism, non-targetable and occurring spontaneously as a consequence of the chemical properties of the metabolites, and including reaction of synthesis, redox, decomposition, replacement and isomerisation analogous to principal enzyme categories [80–82]. For instance, an important contribution of non-enzymatic processes, like oxidation, in the release of seed dormancy related to increased GA levels is well known [80]. Thus, our data suggests that GAc spraying led to different levels of metabolism regulation in the grape inflorescences, resulting in modifications on the levels of amino acids and peptides, nucleotide, carbohydrates, lipids, cofactor and secondary metabolisms, energy production and conversion and signal transduction mechanisms (Additional file 10).

Although the leaf P_n_ values have not been significantly affected by GAc treatment (Table 1), in inflorescences it was observed the up-regulation of two genes encoding photosystem I assembly protein and photosystem II reaction center, as well as three genes encoding RuBisCO, as disclosed by RNA-Seq (Additional file 6). These indicators suggested a global reinforcement of the photosynthetic machinery in inflorescences, what might have been accompanied also by an increase in photorespiration and chlororespiration, since glycolate contents increased accompanied by the up-regulation of seven genes encoding chloroplastic NADH dehydrogenase complex units (Fig. 5). Photorespiration and chlororespiration both involve the oxidation of carbohydrates, the consumption of oxygen and are associated with light energy dissipation [67, 83, 84]. Likewise, respiration seemed be enhanced in GAc treated inflorescences, as revealed by the up-regulation of other 13 genes encoding NADH dehydrogenases, one encoding cytochrome b and two encoding cytochrome c oxidase from respiratory electron transport chain in mitochondria (Fig. 5 and Additional file 6).

These results concerning the photosynthetic and respiration pathways, together with the up-regulation of DEG assigned to sugar and polysaccharide-related pathways, suggested therefore a stimulation of the energy metabolism on inflorescences. This hypothesis is further supported by the accumulation of the precursor ribose, purine and pyrimidine nucleotides (Additional file 9), which play a central role as energy carriers and subunits of nucleic acids, indicating also a global increased in gene expression. Accordingly, the decreased glucose 6-phosphate, fructose-1,6-biphosphate and mannose-6-phosphate (Fig. 5) suggests a degradation of these molecules to generate ribulose-5-phosphate, which is a precursor of the nucleotides synthesis. Furthermore, the overall induction of nucleotide and carbohydrates metabolism in response to GAc used as abscission inducing treatment in grapevine was previously reported [12].

The increased mannitol content is known be related to stress tolerance due to the osmoprotectant function [85]. Only GAc promoted an accumulation of the ascorbate-precursor galactose [86], in agreement with the increase in dehydroascorbate previously reported in a different genetic background [12].

GAc caused changes in the inflorescences levels of transcripts and metabolites involved in the secondary metabolism at bloom (Fig. 5), similarly to what was previously observed in an earlier phenological stage (pre-bloom) [30]. The quercetin-3-O-glucoside and naringenin accumulation and repression of a gene encoding a hyoscyamine 6-dioxygenase suggests induced flavonoids metabolism. In particular, a gene encoding an UDP-glycosyltransferase 74B1, proposed to be involved in the secondary metabolism and as a defense response by callose deposition into the CW, is also part of IAA biosynthetic pathway. Hence, the up-regulation of this gene suggests that the increment of auxin contents might be needed for the GAc-induced responses [29]. On the other hand, taking into account the down-regulation of *GIBBERELLIN 3-BETA-DIOXYGENASE 1* and the increased GA_8_ content which results from GA_1_ inactivation (Fig. 5 and Table 5), a reduction of the endogenous bioactive GA level can be suggested, probably due to a negative-feedback regulation promoted by GAc spraying, as previously observed after GAc treatments in different phenological stages [29, 30]. An auxin regulation of bioactive GAc levels has been suggested, corroborating this assumption [87].

### Shade induced abscission by nutritional stress and global metabolism repression

'Thompson Seedless' showed to be sensitive to shade imposed at 50 % cap fall and during 14 days, resulting in increased flower drop percentages (Table 1). These observations at bloom stage together with depicted less compact bunches and lower number of berries at harvest (Table 2), suggests that this approach can be exploited as an alternative method for thinning berries. The observed decline of P_n_ to zero will consequently decrease C-resources available to both vegetative and reproductive sinks. This will increase the competition between sinks [88–90], and promote flower abortion [17]. Our results highlighted also the importance of the P_n_ during bloom to the developing cluster, despite the carbohydrate reserves [91]. Shading also affected leaf chlorophyll content, total leaf area and shoot growth (Table 1), showing a more pronounced effect in vegetative growth comparing to previously observations under greenhouse conditions [12]. This might have been related to a higher percentage of light intercepted, to the different genetic background and to the field growing conditions, in the present work.

Shade resulted in the down-regulation of a large group of genes involved in photosynthesis, carbohydrates metabolism and transport [18, 92], and reduced carbon and carbon derived metabolites content [12, 15] (Additional file 6 and Additional file 9). The accumulation of arabonate and xylonate were the only exceptions at the metabolite level. These monosaccharides decorate CW polymers, such as pectins or xyloglucans, and its presence can result from CW remodeling processes that occur during pedicel AZ formation, protective layer differentiation on the proximal side after organ detachment [93] and alterations on CW structure and growth in adaptation to the imposed abiotic stress [94]. Their accumulation is also in accordance to the differentially expression of pectinesterases (EC 3.1.1.11), polygalacturonases (EC 3.2.1.15), expansins, cellulose synthase (EC 2.4.1.12) and callose synthase (EC 2.4.1.34). Consistently, starch and sucrose metabolism was the most represented affected pathway which is reported to be very sensitive to environment changes, providing the mobilization of stored carbohydrates [95] (Additional file 11). The analysis of the impact of shade on sugar signaling pathway and transport (Table 7) showed that expression of genes from Sucrose Nonfermenting-1 (SNF1) Related protein Kinases1(SnRK1) family was significantly affected, which were identified as central regulators of the transcriptome in response to darkness and multiple types of stress signals triggering extensive transcriptional changes [96]. The predominant up-regulation of trehalose-6-phosphate synthase (EC 2.4.1.15) genes is also involved in SnRK1 signaling [97]. Hexose kinases and invertases (EC 3.2.1.26, 3.2.1.48), involved in sugar signaling [98, 99], showed to be implicated in organ abscission *via* shading, as previously reported [18]. Sucrose mobilization was also induced during shade, through the up-regulation of a gene encoding the reversible sucrose synthase (EC 2.4.1.13), indicating altered sucrose and sucrose-derived metabolites and sucrose-specific signaling pathway [100].

At transcriptomic and metabolomic levels, shade imposition led to a classic signature of carbon/nitrogen (C/N) imbalance due to carbon deficit, with a stimulation of amino acids metabolism, a repression of energy metabolites and carbon metabolites pathways and increased accumulation of oxidative stress markers [95, 101]. According to the amino acid and peptide biosynthesis, metabolism and transport associated pathways affected by the shade treatment, the increased content of the proteinogenic amino acids may result from amino acid biosynthesis and from enhanced protein turnover to free up amino acid carbon backbones for energy utilization. Particularly, the increased aromatic amino acids phenylalanine, tyrosine and tryptophan contents might result from stress- induced protein breakdown, as revealed by the decline of the biosynthetic precursor shikimate levels, simultaneous with the down-regulation of genes encoding enzymes of the shikimate pathway, as shikimate kinase (EC 2.7.1.71) and the bi-functional enzyme 3-dehydroquinate dehydratase/shikimate 5-dehydrogenase (EC 4.2.1.10) (Additional file 11). Our results are in accordance with previous studies which demonstrate that abiotic stresses enhance accumulation of beatine, proline and allantoin [95, 102, 103]. Allantoin, which was the mostly increased metabolite in inflorescences developing under shade (Additional file 9), often accumulates as a response to C/N imbalances, and results from purine degradation is implicated in nitrogen metabolism and stress tolerance by activation of abscisic acid metabolism [104].

Our data suggests that, under shade imposition, ABA biosynthesis, catabolism and signaling pathways were stimulated (Table 8). The effect of ABA in abscission can be directly related to the activation of ABA-signaling genes and/or indirectly associated to an ACC increase and to ethylene biosynthesis [8, 11]. On the other hand, ethylene accumulation can promote ABA catabolism as a consequence of increased ABA 8′-hydroxylase activity [105], which resulted in a reduced net ABA content (Table 5). The decreased ABA content was also observed by [106], as response from the soybean reproductive structures to shading.

Ethylene-auxin balance is recognized as one of the most important regulators of organ abscission determination [9, 48]. The acquisition of sensitivity to ethylene by the AZ cells has been associated with an altered expression of auxin-regulated genes as a result of auxin depletion [7]. Moreover, [48] showed that auxin regulates the timing of organ abscission and that a functional IAA signaling pathway is required for setting up the event. Our data shows that auxin biosynthesis was induced in shaded-treated inflorescences, and the auxin signaling pathway was active with the up-regulation of genes encoding auxin receptors TIR1 and ABP and down-regulation of *Aux/IAA* and *ARF* genes in both time points investigated (Table 8). On the other hand, the up-regulation of a gene encoding an IAA-amido synthetase GH3.9 only at 5d, as previously observed in shade-induced lychee abscission [92], indicated that auxin conjugation reducing the free IAA content, can exert an important role in auxin-ethylene balance. Accordingly, auxin transport showed to be repressed by down-regulation of *AEC* genes only at 7d, as previously reported [18] in response to the fruit abscission induction by naphthaleneacetic acid application.

Ethylene biosynthesis and signal transduction pathways were induced in shade treated inflorescences, involving the accumulation of cyano-alanine and the predominantly up-regulation of genes encoding ACC oxidases and *EIN3* and differentially regulation of ERF family of transcription factors involved in activation or repression of transcription activity [3, 107] (Additional file 9 and Table 8). In particular key elements of MAPK cascades related to ABA and ethylene signal transduction pathways [108], and known to be involved in floral organ abscission [20] were regulated, as those coding for MAPK3, MAPK4, MAPKK5 and MAPKK6 (Table 8). In addition, GTPase mediated signal transduction, upstream of MAPK cascades [109], was induced during shade treatment (Table 6) and was previously shown to be involved in leaf abscission signaling and ethylene biosynthesis [110] and to regulate the movement of key molecules required for abscission [111]. GAs biosynthetic and signaling pathways were predominantly repressed, accordingly with [112] that demonstrated that fruit abscission is enhanced by low carbohydrates and GAs availability.

The significant impact on CKs activation, perception and degradation caused by light reduction during bloom (Table 8) highlights the role of this hormones class, and is in accordance with the described CKs action as abscission-accelerating signal [11], although following the hypothesis of having ethylene regulation [113]. Also BR, SA and JA metabolisms were correlated with the abscission boost caused by shade (Table 8). The accumulation of SA content agrees with the down-regulation of genes encoding salicylate-O-methyltransferase and the general up-regulation of genes encoding PALs (Additional file 6) involved in its own biosynthesis [114]. In addition, the accumulation of oxidized lipids confirmed to occur in response to shade, as 13-HODE and 9-HODE, products of elevated oxidative status, have been linked to the JA biosynthetic pathway [115].

Some of the most striking changes observed in shaded inflorescence samples were represented by DEG and accumulation of metabolites associated with oxygen stress remediation (Table 9 and Additional file 9), and amongst them, the intermediates of the glutathione synthesis cycle were the most represented, as previously reported [12]. On the other hand, ascorbate metabolism seemed to be inhibited, as suggested by the down-regulation of genes encoding ascorbate oxidase, ascorbate peroxidase and GDP-l-galactose phosphorylase, concomitantly with decreased levels of metabolites related with ascorbate metabolism. Regarding secondary metabolism, the fact that flavonoids and diterpenoids-related pathways had been predominantly repressed, while phenylpropanoids and stilbenoid-related pathways were predominantly induced (Additional file 6), pinpoints a slowdown of biochemical reactions while promoting the activation of stress responses and defense systems during abscission [116].

Among the transcription factors differentially regulated by shade treatment, members of MADs-box, AP2, MYB, WRKY, zinc finger transcription factor families were previously described to participate in abscission regulation [7, 11, 18, 92, 117].

## Conclusions

The two imposed treatments induced flower abscission by exerting different effects on grapevine inflorescences metabolism, agreeing with the mechanistic model previously proposed [12]. GAc treatment response suggested a reinforcement of the energetic metabolism simultaneously with induction of nucleotide biosynthesis and carbon metabolism. A global metabolism stimulation of the central flower (king flower), which open before the smaller lateral ones [33], by GAc application, can be hypothesize, promoting the fruit set of these flowers and the developmental inhibition and abscission of the later ones. On the other hand, shade imposition induced carbohydrate metabolism repression, promoting flower drop by the previously described abscission process *via* nutritional stress [11, 18] associated with sugar-, ethylene- and auxin-responsive signaling pathways and other signaling pathways to coordinate abscission. Regulation of polyamines metabolism, activation of ROS scavenging mechanisms, alterations on ethylene signaling pathway and bioactive GA biosynthesis repression were identified as candidate common signatures of abscission. Our data provided a new insight on alternative pathways leading to abscission, which can assist the development and optimization of strategies for abscission control in fruit crop species.

## Availability of supporting data

The data sets supporting the results of this article have been submitted to the Sequence Read Archive at NCBI (http://www.ncbi.nlm.nih.gov/sra) under accession numbers [SRA:SRX964421, SRX964430, SRX966723, SRX966735, SRX966740, SRX966742, SRX966755, SRX1008101, SRX1008174, SRX1008177, SRX1008181, SRX1008185, SRX1008211, SRX1008213, SRX1008214, SRX1008217, SRX1010114, SRX1010115].

## Additional files

Additional file 1: Figure S1. Microclimate conditions recorded during bloom period (twelve days) under shaded and unshaded conditions. Mean values per hour of relative humidity (RH), temperature and photosynthetic photon flux density (PPFD) (±se). (PDF 155 kb) Additional file 2: Figure S2. Quantitative rtPCR validation of the RNA-seq. NCBI reference, gene identification (GID), fold change quantification by q-rtPCR and RNA seq, primers and amplicon length (bp) for abscission related-genes (A). NCBI reference, primers and amplicon length are also given for references genes (B). Data are means of 3 replicates. The pearson correlation coefficient between qRT-PCR and RNA-Seq obtained fold-changes (R = 0.84) is significant at *p*-value ≤ 0.001 (C). (PDF 176 kb) Additional file 3: Table S1. Reference library of chemical standard used for LC/MS/MS analysis. (XLSX 28 kb) Additional file 4: Table S2. Parameters for hormone identification in Mass Spectrometry. Cone voltage potential, collision energy (CE) and other performance characteristics. (PDF 67 kb) Additional file 5: Figure S3. Pie charts summarizing the results of alignment of each sample against the *Vitis vinifera* genome. Percentage of reads by sample, mapped uniquely (black), unmapped (grey) or mapped in multiple locations (white). (PDF 54 kb) Additional file 6: Table S3. List of genes significantly affected by GAc and shade treatments. Gene annotation, functional categories and respective fold-change. The abbreviations GAc5d, GAc7d, SH5d and SH7d mean the log2 fold-change between gene relative expression obtained in treated and control inflorescences, at 5 and 7 days after 100 % cap fall. (XLSX 777 kb) Additional file 7: Figure S4. Orthogonal Signal Correction Partial Least Squares Discriminant Analysis (O-PLS-DA) of differential expressed genes (A) and differentially accumulated metabolites (B). The genes and metabolites are grouped and the dispersion color was coded by their KOG categories or super-pathway, respectively. Data was ln-transformed prior to the analyses. (PDF 140 kb) Additional file 8: Figure S5. Pearson correlation plots of RNA-Seq reads. Correlation between individual biological replicates in each time-point (5 and 7d) and treatment (control, GAc and shade) using ln-transformed read counts for the DEG as input. All correlation values are significant at *p*-value ≤ 0.001. (PDF 140 kb) Additional file 9: Table S4. Heat map illustrating metabolite changes in response to GAc and shade treatments. The abbreviations GAc5d, GAc7d, SH5d and SH7d mean the log2 fold-change between metabolite relative content obtained in treated and control inflorescences, at 5 and 7 days after 100 % cap fall. Red and green shaded cells indicate *p*-value ≤ 0.05. Light red and light green shaded cells indicate 0.05 < *p*-values ≤ 0.10. (XLSX 70 kb) Additional file 10: Figure S6. Distribution of differentially changed transcripts and metabolites according to functional categories. Each pie chart corresponds to changes occurred as response to GAc and shade treatments at 5 and 7 days after 100 % cap fall (d). (PDF 234 kb) Additional file 11: Table S5. Enzymatic classification of differentially expressed genes and respective fold-change for each treatment at 5 and 7d. (XLSX 68 kb) Additional file 12: Table S6. List of GO enriched categories in each pair-wise comparison. Enrichment analysis was performed using R bioconductor topGO package with Fisher's exact test and *p*-value ≤ 0.01. (XLSX 56 kb) Additional file 13: Figure S7. Acyclic graphs with the mostly enriched GO categories in differentially expressed genes. The top 5 and top 5-related biological processes, molecular function and cellular component at GAc 7d (A), SH5d (B) and SH7d(C) are shown. The color scale presented is related with the *p*-value, where the redder the node, lower the *p*-value. The nodes with black outline are the top 5 enriched categories. (PDF 440 kb)

## Abbreviations

ABA: Abscisic acid
ACC: 1-aminocyclopropane-1-carboxylate
ARF: Auxin response factor
AZ: Abscission zone
BR: Brassinosteroid
CK: Ccytokinin
CW: Cell wall
DEG: Differentially expressed genes
d: Days after 100 % cap fall
ERF: Ethylene response factor
IAA: Indole-3-acetic acid
FPKM: Fragments per kilobase of exon per Million fragments mapped
GA: Gibberellin
GAc: Gibberellic acid
GA20ox: Gibberellin20 oxidase
GO: Gene Ontology
*g*_*s*_: Stomatal conductance
JA: Jasmonic acid
KEG: Kyoto Encyclopedia of Genes and Genomes
KOG: euKaryotic Orthologous Groups
MAPK: Mitogen activated protein kinase
PA: Polyamine
PAL: Phenylalanine ammonia-lyase
PCD: Programmed cell death
P_n_: Net photosynthetic rate
PMN: Plant Metabolic Network
RNA-Seq: Ribonucleic acid sequencing
ROS: Reactive oxygen species
RuBisCO: Ribulose-1,5-bisphosphate carboxylase-oxygenase
SA: Salicylic acid
SAM: S-adenosylmethionine
TCA: Tricarboxylic acid
