# Supplementary material for: Shared and divergent pathways for flower abscission are triggered by gibberellic acid and carbon starvation in seedless Vitis vinifera L
Source: BMC Plant Biol. 2016 Feb 1;16:38. doi: 10.1186/s12870-016-0722-7 (PMC4736245; doi:10.1186/s12870-016-0722-7)
Supplement: Additional file 1: Figure S1. — Microclimate conditions recorded during bloom period (twelve days) under shaded and unshaded conditions. Mean values per hour of relative humidity (RH), temperature and photosynthetic photon flux density (PPFD) (±se). (PDF 155 kb) [file 12870_2016_722_MOESM1_ESM.pdf]

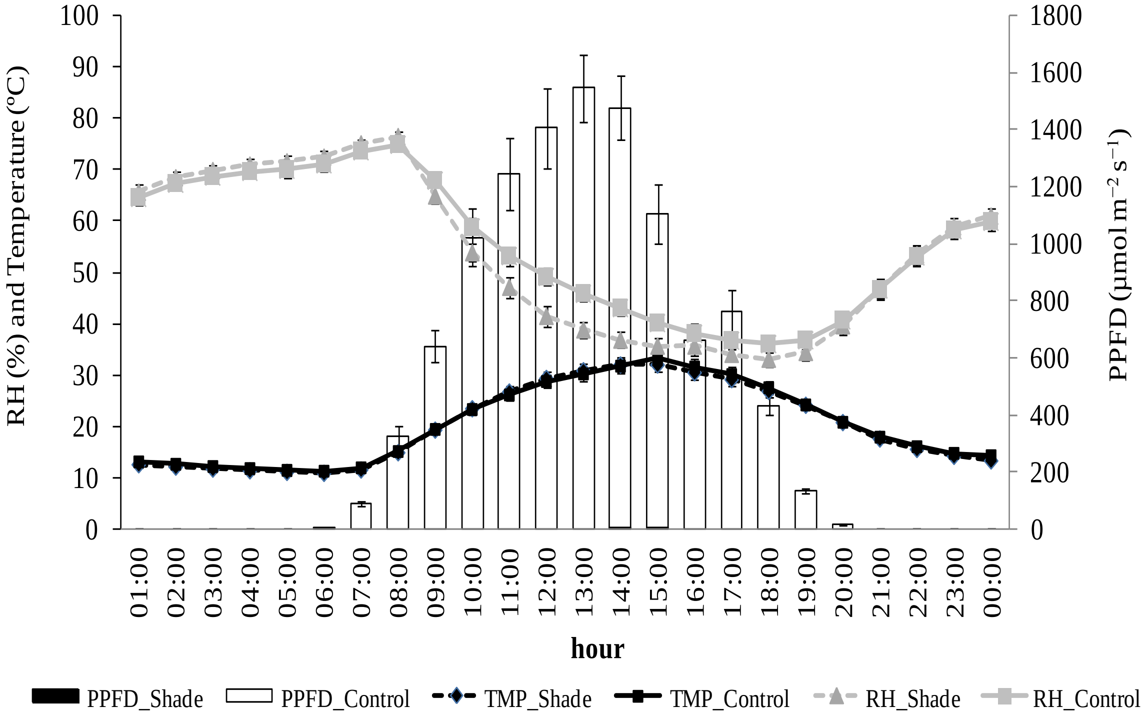

Additional file 1. Figure S1. Microclimate conditions recorded during bloom period (twelve days) under shaded and unshaded conditions. Mean values per hour of relative humidity (RH), temperature and photosynthetic photon flux density (PPFD) (±se).
