# Supplementary material for: Shared and divergent pathways for flower abscission are triggered by gibberellic acid and carbon starvation in seedless Vitis vinifera L
Source: BMC Plant Biol. 2016 Feb 1;16:38. doi: 10.1186/s12870-016-0722-7 (PMC4736245; doi:10.1186/s12870-016-0722-7)

Additional file 2. Figure S2. Quantitative rtPCR validation of the RNA-seq. NCBI reference, gene identification (GID), fold change quantification by q-rtPCR and RNA seq, primers and amplicon length (bp) for abscission related-genes (A). NCBI reference, primers and amplicon length are also given for references genes (B) . Data are means of 3 replicates. The pearson correlation coefficient between qRT-PCR and RNA-Seq obtained fold-changes (R= 0.84) is significant at *p*-value≤0.001 (C).

A

NCBI	GID	description	Fold change (q-rtPCR)				Fold change (RNAseq)				primer forward (5'-3')	primer reverse (5'-3')	bp
			GAcT5	GAcT7	SHT5	SHT7	GAcT5	GAcT7	SHT5	SHT7			
XM_002276122.1	VIT_04s0023g02420	mitogen-activated protein kinase 4	0.36	0.41	**1.60**	**2.68**	0.65	0.25	0.97	**1.51**	TATTATCAAGTCCCCGCAGCC	GAGGTTCCCAGGCTTCAAGTC	127
XM_002273277.2	VIT_05s0020g02910	mitogen-activated protein kinase kinase 5	-0.10	-0.34	-0.47	**-2.09**	0.00	0.11	-0.43	**-3.10**	AGGAGGTTGCTGCTCTCTTCC	GCAGGCCTCAAGTTTGGTTCC	127
XM_002284983.2	VIT_06s0004g03130	auxin response factor 4	0.21	-0.12	-0.37	**-2.39**	-0.56	-0.19	-0.60	**-2.52**	GCCAAGGCGACATCTGCTTAC	AGCTCTCCACCTTCACCTCTC	103
XM_002284771.1	VIT_06s0004g03540	mitogen-activated protein kinase 3	-0.50	0.07	**1.60**	**2.29**	-0.24	0.21	1.32	**2.10**	CAGAAGGCCTTTATTTGCGGG	TCCGAACAAACCCAAGATCAG	99
XM_002283455.2	VIT_11s0016g02970	mitogen-activated protein kinase kinase 6	0.73	-0.11	-0.01	-0.17	-0.34	0.44	-0.62	**-2.24**	GACCTCGTGAGTCACCCTTTC	AGGAGGTTCCAAGCTGCCTAC	84
XM_002266023.1	VIT_11s0052g00440	auxin efflux carrier component 2	0.55	-1.44	**-2.14**	-1.48	0.55	-0.20	-0.55	**-1.92**	ATCGATCAGGACTCAGGCAGC	GTCATGACGCTTGTTGGAGGC	99
XM_002276344.1	VIT_13s0047g00250	ethylene insensitive 3-like	0.41	0.22	1.43	**2.31**	-0.15	-0.11	0.71	**1.66**	GACTGCCAAAGAGAGTGCCAC	AAAGATCCACTCCCACCAGCC	117
XM_003634159.1	VIT_17s0000g02420	auxin efflux carrier component 1	-0.22	-0.29	0.16	-0.96	-0.34	-0.36	-0.55	**-1.67**	TTGTCGTCTACCACTCCACGG	CCACCCGCTACCATTGAGTAG	131

B

NCBI	description	primer forward (5'-3')	primer reverse (5'-3')	bp
XM_002282480.3	actin 1	CTTCCAGCCATCTCTCATTGG	TGTTGCCATAGAGGTCCTTCC	107
XM_002263109.2	glyceraldehyde-3-phosphate dehydrogenase	GGAATAGCACTCAACGAGAAG	TGCCATGTGGACAATCAAGTC	99
M_002282083.2	polyubiquitin	TGGGTCTCAGCCATTTGAAAG	GCCTCACTAACGACCACTTAG	111

C

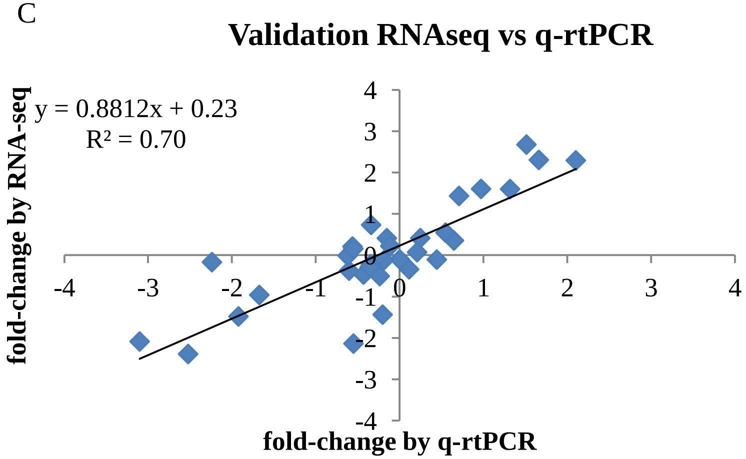

Supplement: Additional file 2: Figure S2. — Quantitative rtPCR validation of the RNA-seq. NCBI reference, gene identification (GID), fold change quantification by q-rtPCR and RNA seq, primers and amplicon length (bp) for abscission related-genes (A). NCBI reference, primers and amplicon length are also given for references genes (B). Data are means of 3 replicates. The pearson correlation coefficient between qRT-PCR and RNA-Seq obtained fold-changes (R = 0.84) is significant at p-value ≤ 0.001 (C). (PDF 176 kb) [file 12870_2016_722_MOESM2_ESM.pdf]
