# Supplementary material for: Shared and divergent pathways for flower abscission are triggered by gibberellic acid and carbon starvation in seedless Vitis vinifera L
Source: BMC Plant Biol. 2016 Feb 1;16:38. doi: 10.1186/s12870-016-0722-7 (PMC4736245; doi:10.1186/s12870-016-0722-7)
Supplement: Additional file 4: Table S2. — Parameters for hormone identification in Mass Spectrometry. Cone voltage potential, collision energy (CE) and other performance characteristics. (PDF 67 kb) [file 12870_2016_722_MOESM4_ESM.pdf]

Additional file 4. Table S2. Parameters for hormone identification in Mass Spectrometry. Cone voltage potential, collision energy (CE) and other performance characteristics.

	**RT (min)**	**Dection Limit (µg/L)**	**Ion mode**	**Cone Voltage (V)**	**Quantifying trace CE (eV)**	**1° qualifying trace CE (eV)**	**2° qualifying trace CE (eV)**
GA1	4.01	5	ESI-	40	347>229; (30)	347>259; (20)	347>273; (30)
GA3	3.95	5	ESI-	36	345>143; (38)	345>221; (24)	345>239; (14)
GA4	6.18	5	ESI-	44	331>289; (24)	331>213; (32)	331>243; (22)
GA8	3.03	5	ESI-	35	363>257; (20)	363>275; (20)	
GA9	6.62	5	ESI-	40	315>227; (20)	315>253; (25)	315>271; (20)
GA12	7.37	5	ESI-	50	331>201; (35)	331>269; (30)	331>287; (25)
GA20	5.24	5	ESI-	40	331>147; (32)	331>173; (34)	331>243; (18)
GA34	5.74	5	ESI-	34	347>199; (30)	347>241; (20)	347>259; (20)
GA53	6.30	5	ESI-	55	347>121; (40)	347>189; (36)	347>233; (32)
ABA	5.00	5	ESI-	24	263>153; (10)	263>201; (16)	263>204; (18)
IAA	4.30	5	ESI+	22	176>77; (34)	176>103; (28)	176>130; (15)

Multiple reaction monitoring mode in negative ion mode (capillary voltage, 1.08 kV) and with argon (0.20 mL/min; gas) and nitrogen (1000 L/h) as collision and desolvation gas, respectively.
