# Supplementary material for: Shared and divergent pathways for flower abscission are triggered by gibberellic acid and carbon starvation in seedless Vitis vinifera L
Source: BMC Plant Biol. 2016 Feb 1;16:38. doi: 10.1186/s12870-016-0722-7 (PMC4736245; doi:10.1186/s12870-016-0722-7)
Supplement: Additional file 5: Figure S3. — Pie charts summarizing the results of alignment of each sample against the Vitis vinifera genome. Percentage of reads by sample, mapped uniquely (black), unmapped (grey) or mapped in multiple locations (white). (PDF 54 kb) [file 12870_2016_722_MOESM5_ESM.pdf]

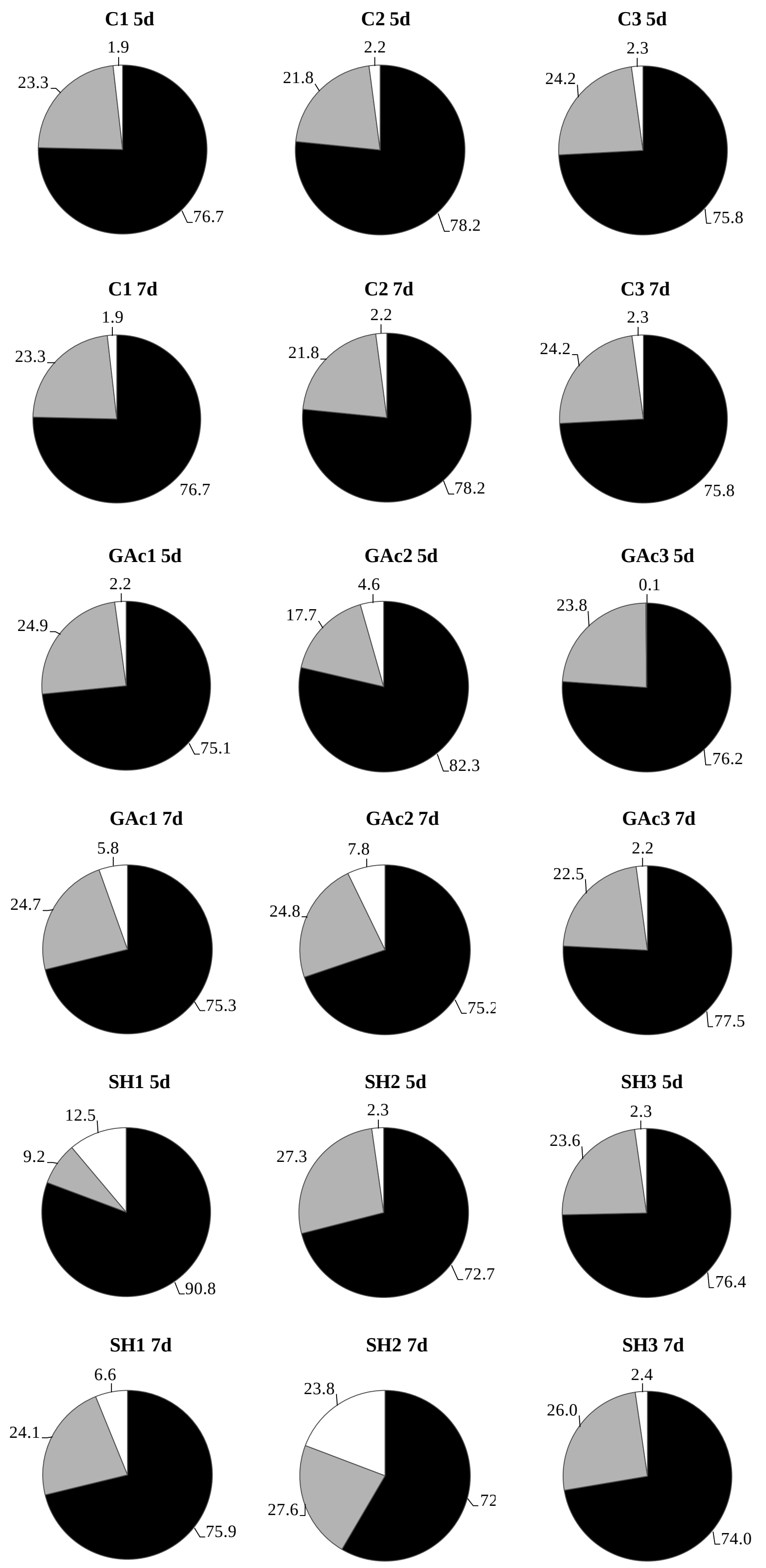

Additional file 5. Figure S3. Pie charts summarizing the results of alignment of each sample against the *Vitis vinifera* genome. Percentage of reads by sample, mapped uniquely (black), unmapped (grey) or mapped in multiple locations (white).
