# Supplementary material for: Shared and divergent pathways for flower abscission are triggered by gibberellic acid and carbon starvation in seedless Vitis vinifera L
Source: BMC Plant Biol. 2016 Feb 1;16:38. doi: 10.1186/s12870-016-0722-7 (PMC4736245; doi:10.1186/s12870-016-0722-7)
Supplement: Additional file 7: Figure S4. — Orthogonal Signal Correction Partial Least Squares Discriminant Analysis (O-PLS-DA) of differential expressed genes (A) and differentially accumulated metabolites (B). The genes and metabolites are grouped and the dispersion color was coded by their KOG categories or super-pathway, respectively. Data was ln-transformed prior to the analyses. (PDF 140 kb) [file 12870_2016_722_MOESM7_ESM.pdf]

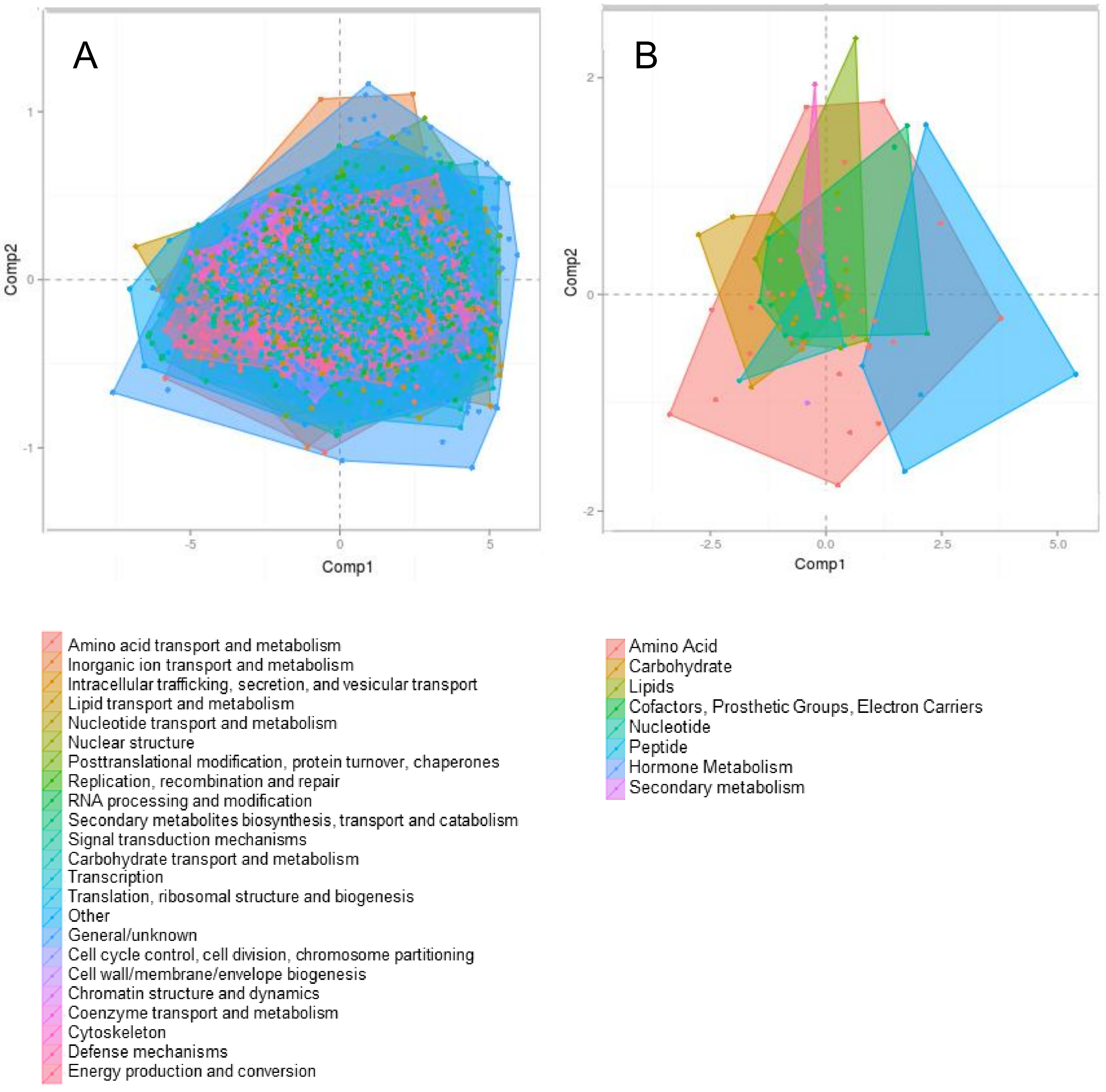

Additional file 7. Figure S4. Orthogonal Signal Correction Partial Least Squares Discriminant Analysis (OPLS-DA) of differential expressed genes (A) and differentially accumulated metabolites (B). The genes and metabolites are grouped and the dispersion color was coded by their KOG categories or super-pathway, respectively. Data was ln-transformed prior to the analyses.
