# Supplementary material for: Shared and divergent pathways for flower abscission are triggered by gibberellic acid and carbon starvation in seedless Vitis vinifera L
Source: BMC Plant Biol. 2016 Feb 1;16:38. doi: 10.1186/s12870-016-0722-7 (PMC4736245; doi:10.1186/s12870-016-0722-7)
Supplement: Additional file 8: Figure S5. — Pearson correlation plots of RNA-Seq reads. Correlation between individual biological replicates in each time-point (5 and 7d) and treatment (control, GAc and shade) using ln-transformed read counts for the DEG as input. All correlation values are significant at p-value ≤ 0.001. (PDF 140 kb) [file 12870_2016_722_MOESM8_ESM.pdf]

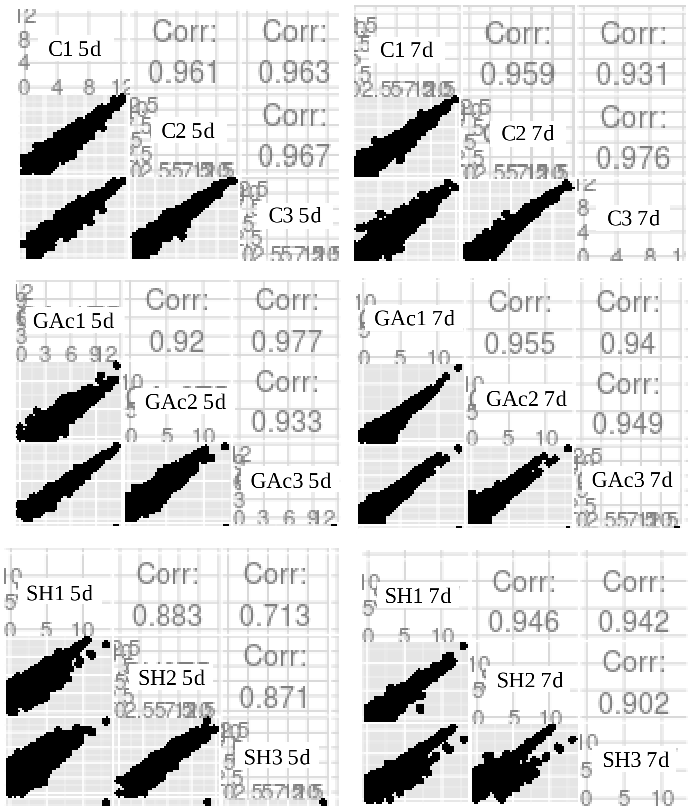

Additional file 8. Figure S5. Pearson correlation plots of RNA-Seq reads. Correlation between individual biological replicates in each time-point (5 and 7d) and treatment (control, GAc and shade) using ln-transformed read counts for the DEG as input. All correlation values are significant at *p*-value≤0.001.
