# Supplementary material for: Shared and divergent pathways for flower abscission are triggered by gibberellic acid and carbon starvation in seedless Vitis vinifera L
Source: BMC Plant Biol. 2016 Feb 1;16:38. doi: 10.1186/s12870-016-0722-7 (PMC4736245; doi:10.1186/s12870-016-0722-7)
Supplement: Additional file 10: Figure S6. — Distribution of differentially changed transcripts and metabolites according to functional categories. Each pie chart corresponds to changes occurred as response to GAc and shade treatments at 5 and 7 days after 100 % cap fall (d). (PDF 234 kb) [file 12870_2016_722_MOESM10_ESM.pdf]

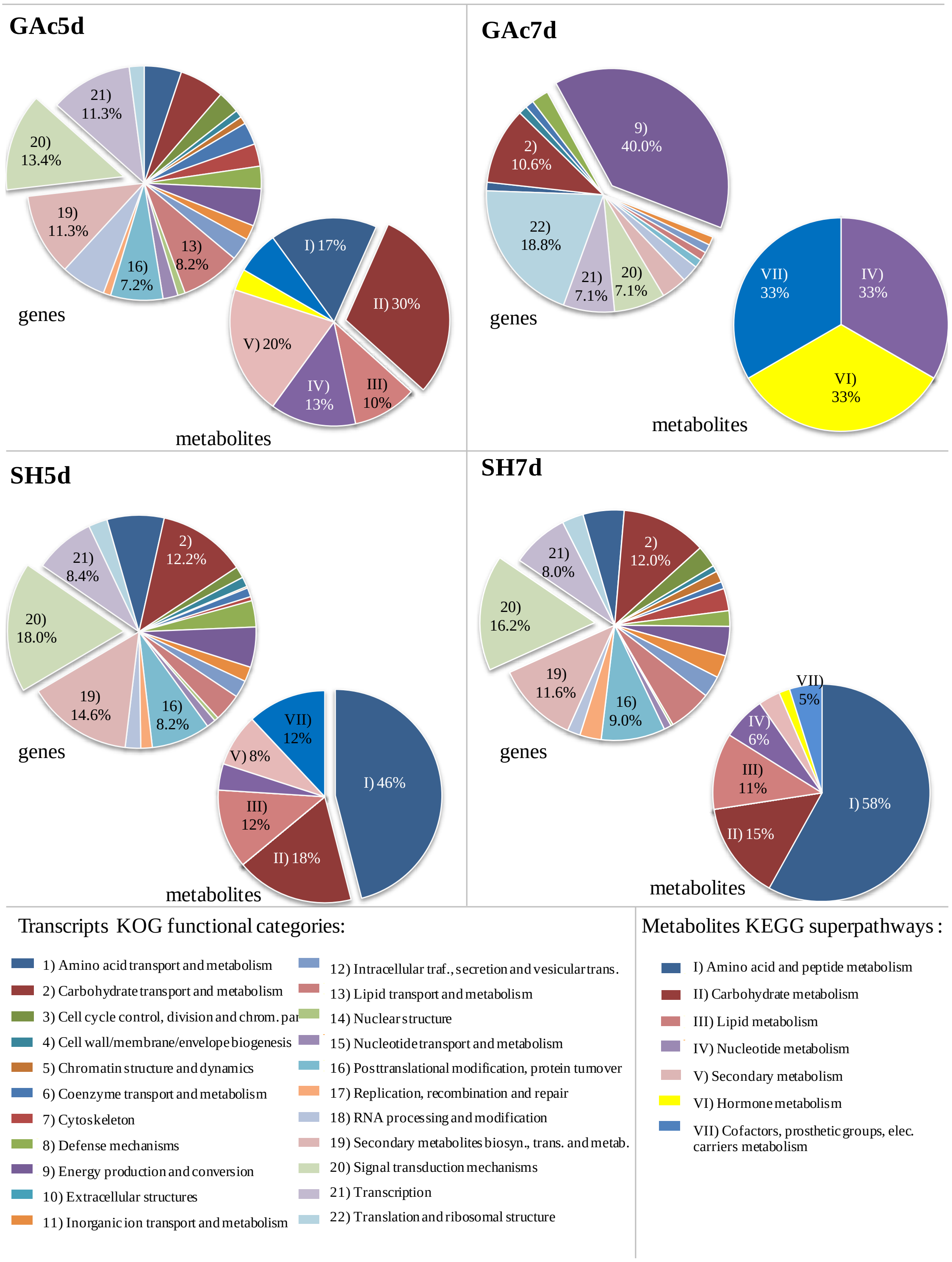

Additional file 10. Figure S6. Distribution of differentially changed transcripts and metabolites according to functional categories. Each pie chart corresponds to changes occurred as response to GAc and shade treatments at 5 and 7days after 100% cap fall (d).
