# Supplementary material for: Shared and divergent pathways for flower abscission are triggered by gibberellic acid and carbon starvation in seedless Vitis vinifera L
Source: BMC Plant Biol. 2016 Feb 1;16:38. doi: 10.1186/s12870-016-0722-7 (PMC4736245; doi:10.1186/s12870-016-0722-7)
Supplement: Additional file 13: Figure S7. — Acyclic graphs with the mostly enriched GO categories in differentially expressed genes. The top 5 and top 5-related biological processes, molecular function and cellular component at GAc 7d (A), SH5d (B) and SH7d(C) are shown. The color scale presented is related with the p-value, where the redder the node, lower the p-value. The nodes with black outline are the top 5 enriched categories. (PDF 440 kb) [file 12870_2016_722_MOESM13_ESM.pdf]

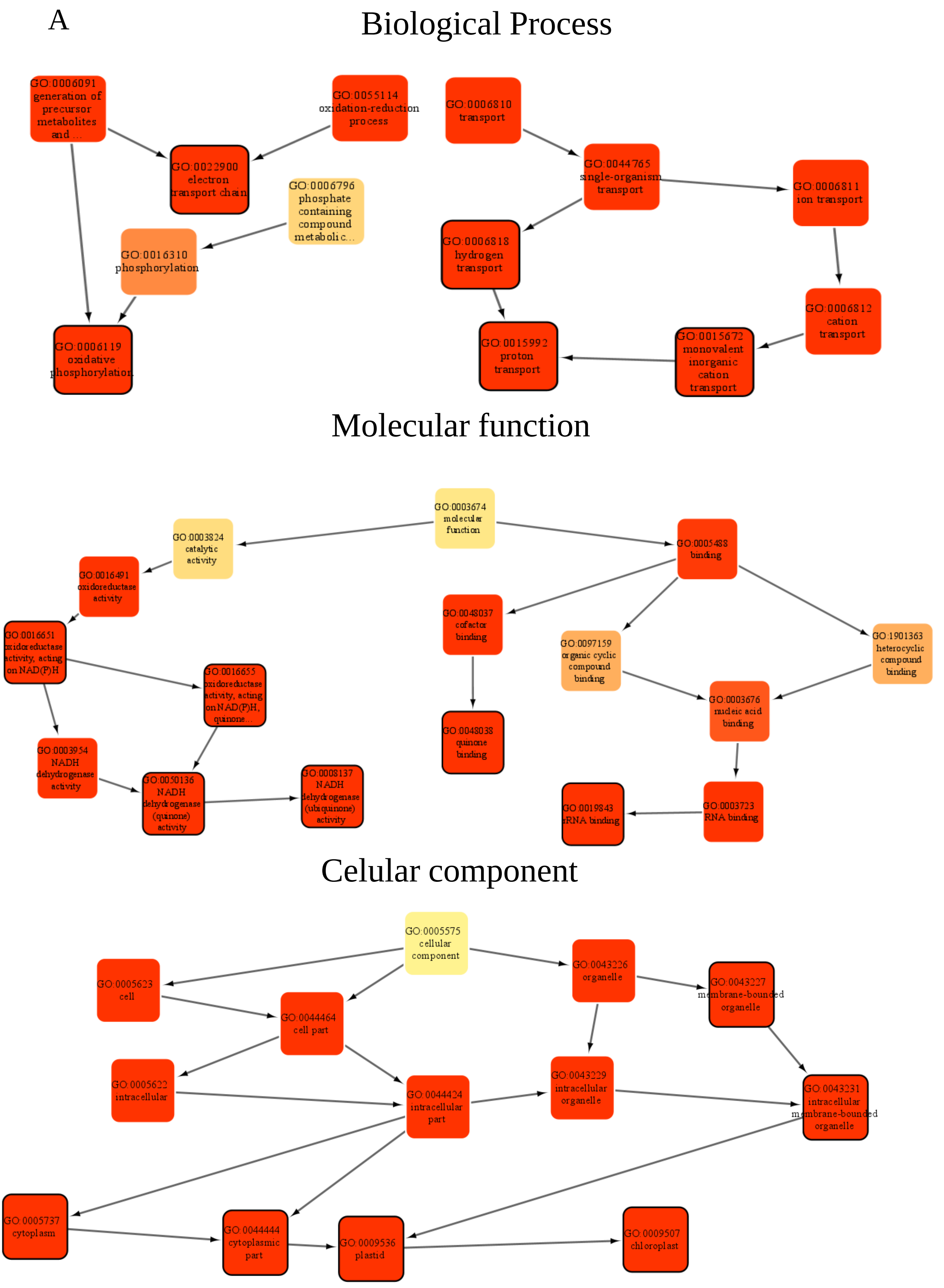

Additional file 13. Figure S7. Acyclic graphs with the mostly enriched GO categories in differentially expressed genes. A) The top 5 and top 5-related biological processes, molecular function and cellular component at GAc 7d are shown. The color scale presented is related with the *p*-value, where the redder the node, lower the *p*-value. The nodes with black outline are the top 5 enriched categories.

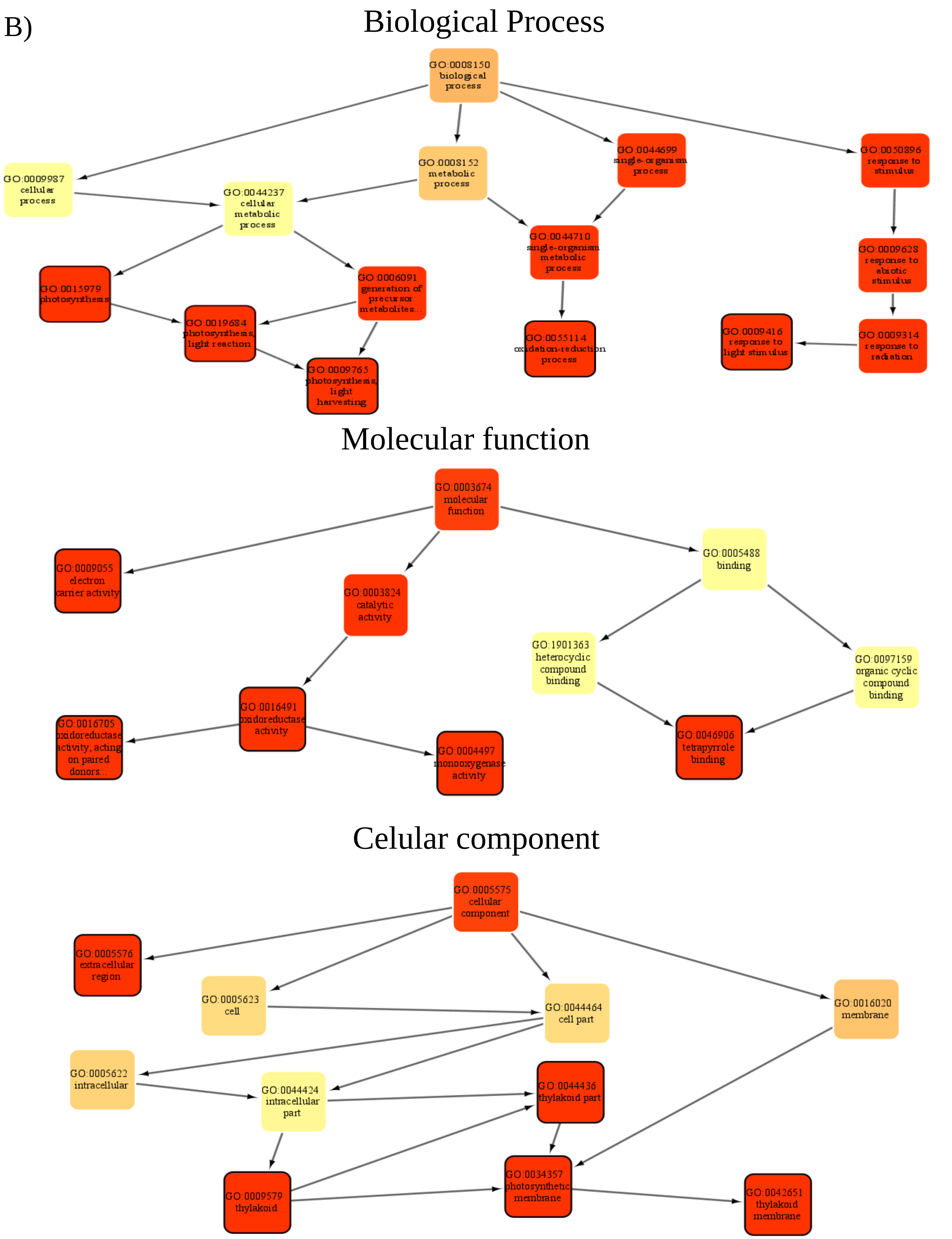

Additional file 13. Figure S7. Acyclic graphs with the mostly enriched GO categories in differentially expressed genes. B) The top 5 and top 5-related biological processes, molecular function and cellular component at SH 5d are shown. The color scale presented is related with the *p*-value, where the redder the node, lower the *p*-value. The nodes with black outline are the top 5 enriched categories.

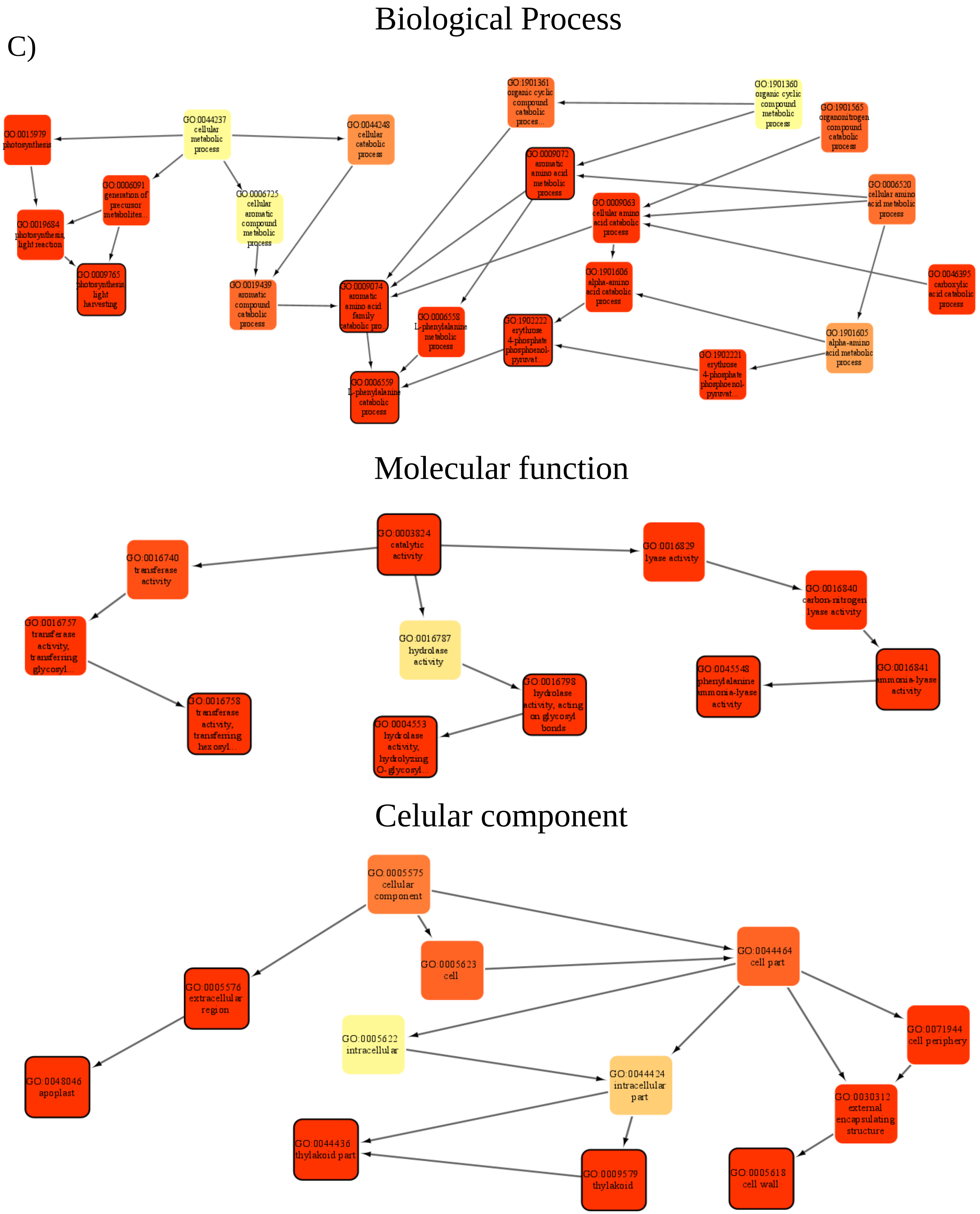

Additional file 13. Figure S7. Acyclic graphs with the mostly enriched GO categories in differentially expressed genes. C) The top 5 and top 5-related biological processes, molecular function and cellular component at SH7d are shown. The color scale presented is related with the *p*-value, where the redder the node, lower the *p*-value. The nodes with black outline are the top 5 enriched categories.
